# {Zn^II^_2_} and {Zn^II^Au^I^} Metal Complexes with Schiff Base Ligands as Potential Antitumor Agents Against Human Glioblastoma Multiforme Cells

**DOI:** 10.3390/molecules31010173

**Published:** 2026-01-01

**Authors:** Lora Dyakova, Tanya Zhivkova, Abedulkadir Abudalleh, Daniela C. Culita, Teodora Mocanu, Augustin M. Madalan, Anamaria Hanganu, Gabriela Marinescu, Emanuil Naydenov, Radostina Alexandrova

**Affiliations:** 1 Institute of Neurobiology, Bulgarian Academy of Sciences, Acad. Georgi Bonchev Str., Bl. 23, 1113 Sofia, Bulgaria; sigma13@abv.bg; 2 Institute of Experimental Morphology, Pathology and Anthropology with Museum, Bulgarian Academy of Sciences, Acad. Georgi Bonchev Str., Bl. 25, 1113 Sofia, Bulgaria; tani413@abv.bg (T.Z.); alkader78mah@yahoo.com (A.A.); 3 Ilie Murgulescu Institute of Physical Chemistry, Romanian Academy, 202 Splaiul Independentei, 060021 Bucharest, Romania; dculita@icf.ro (D.C.C.); tmocanu@icf.ro (T.M.); 4 Department of Inorganic Chemistry, Organic Chemistry, Biochemistry and Catalysis, Faculty of Chemistry, University of Bucharest, 90–92 Panduri St., 050663 Bucharest, Romania; 5 “C.D. Nenitzescu” Institute of Organic and Supramolecular Chemistry of the Romanian Academy, 202B Splaiul Independentei, 060023 Bucharest, Romania; 6 Department of Neurosurgery, University Hospital St. Ivan Rilski, Blvd. “Acad. Ivan Geshov” 15, 1113 Sofia, Bulgaria; emanuilis@abv.bg

**Keywords:** glioblastoma multiforme, cytotoxic/antiproliferative activity, Zn(II) homometallic complexes, Zn(II)Au(I) heterometallic complexes

## Abstract

The challenges of glioblastoma multiforme treatment are related to limitations in tumor removal surgery, its high heterogeneity and aggressiveness, development of resistance to standard therapy, the blood–brain barrier, and the side and toxic effects of the conventional antitumor agents used in clinical practice. Although new treatment strategies continue to emerge, progress remains slow and has not resulted in substantial improvements in patient survival. The main goal of research in recent years has been aimed at developing ways to deal with all these challenges. One of the ways to improve the control of glioblastomas is the introduction of effective new antitumor agents. Metal complexes represent a particularly promising class of compounds in this context. This is why the aim of this study was to assess the effects of six homo- and heterometallic coordination compounds bearing Schiff base ligands—[Zn_2_(Ampy)(µ-OH)(H_2_O)_2_](ClO_4_)_2_ (**ZnAmpy**), [Zn_2_(Dmen)(µ-OH)(H_2_O)_2_](ClO_4_)_2_ (**ZnDmen**), ^1^_∞_[{Zn_2_(Ampy)(μ_3_-OH)}_2_(H_2_O){μ-[Au(CN)_2_]}](ClO_4_)_3_·THF·H_2_O (**ZnAmpyAu**), [{Zn_2_(Dmen)(μ-OH)}_2_{μ-[Au(CN)_2_]}{[Au(CN)_2_]_2_}](ClO_4_)·H_2_O (**ZnDmenAu**), ^1^_∞_[Zn(Salampy){μ-Au(CN)_2_}] (**ZnSalampyAu**), and ^1^_∞_[Zn(Saldmen)(μ-Au(CN)_2_}] (**ZnSaldmenAu**)—on the viability and proliferation of 8MGBA and U251MG human glioblastoma multiforme cells (HDmen and HAmpy are bicompartmental Schiff base ligands resulting from the condensation of 2,6-diformyl-*p*-cresol with N,N-dimethylethylenediamine and 2-(aminomethyl)pyridine, respectively, while HSaldmen and HSalampy are tridentate Schiff base ligands obtained via condensation of salicylaldehyde with N,N-dimethylethylenediamine and 2-(aminomethyl)pyridine, respectively). Among these compounds, **ZnSaldmenAu** is a new compound and is reported here for the first time. Cytotoxicity of the compounds was evaluated through analysis of cell viability, 2D/3D growth, cytopathological alterations, and induction of cell death. The results obtained by methods with different targets in cells and the associated mechanisms of action revealed that the compounds investigated show promising cytotoxic/potential antitumor activity in treated cells.

## 1. Introduction

Glioblastoma multiforme (GBM) is one of the most common primary brain tumors in humans, accounting for 14.5% of all central nervous system tumors and 48.6% of all central nervous system malignancies. It is also the second most common type of cancer in children, responsible for 3–15% of primary tumors in this age group [1]. GBM is an extremely aggressive, invasive, and heterogeneous tumor characterized by high resistance to available therapy, making it difficult to treat and one of the leading causes of cancer-related death [2]. The World Health Organization (WHO) classifies GBM as a grade IV astrocytoma, which has very poor prognosis, resulting in an approximately 30% one-year survival rate and only about 3–5% of patients surviving beyond 5 years [3]. Despite ongoing advances in treatment strategies, improvements in patient survival remain limited. The current treatment standard includes surgical removal of the tumor followed by radiotherapy and chemotherapy, most commonly with the DNA-alkylating agent temozolomide. However, the efficacy of emerging approaches, such as immunotherapy and targeted therapies in GBM, has thus far been modest [4,5]. The limitations in brain tumor removal surgery are the location of the tumor and risk of significant complications such as intracranial hemorrhage, brain nerve damage, and functional area impairment, as well as the highly invasive nature of the tumor. Residual, invasive cancer cells contribute to tumor recurrence [3,6]. Treatment of glioblastoma is also complicated by the selective structure of the blood–brain barrier (BBB), allowing only a limited number of lipophilic drugs to pass through and reach the tumor at therapeutically effective concentrations.

On the other hand, the development of resistance to standard therapy represents a severe problem in GBM treatment. Clinical studies have shown that approximately half of patients do not respond to temozolomide-based therapy due to high expression of the DNA repair protein *O*^6^-methylguanine-DNA methyltransferase (MGMT) [7]. Another potential source of GBM relapse could be so-called cancer stem-like cells (CSCs). In vitro studies have shown that differentiated GBM cells can regain CSC properties when exposed to continuous temozolomide treatment [8]. This resistance has generated a compelling need for innovative and more effective therapeutic strategies for patients with GBM. Moreover, the genetic and epigenetic variation among glioblastoma cells also makes the development of therapies capable of eradicating all tumor cells challenging and, currently, impossible. An important component of glioblastoma growth is the ability of tumor cells to communicate with and manipulate other cells within the brain environment, thereby promoting tumor progression and drug resistance. The vasculogenic mimicry capacity of gliomas (the ability of GBM cells to form vessel-like structures without the help of endothelial cells to supply the tumor with blood) is also considered an obstacle to overcoming the disease [9].

The main goal of research in recent years has been aimed at developing ways to deal with the multiple challenges associated with glioblastoma treatment, including the drug resistance of tumor cells, their escape from the immune response, tumor heterogeneity, limited access of drugs to the tumor due to the blood–brain barrier, and side and toxic effects of conventional antitumor agents used in clinical practice [10,11]. One promising approach to improve the control of glioblastomas is the introduction of effective new antitumor agents, among which metal complexes have shown considerable potential.

Metal-based compounds, especially zinc and gold complexes, have recently gained increasing attention in the design of cancer therapeutics. This interest was initially sparked by the introduction of the first metal-based antitumor agent into clinical oncology, cisplatin (*cis*-diaminodichloro-platinum(II), *cis*-[PtCl_2_(NH_3_)_2_], *cis*-DDP) [12]. Due to their biological activities, there are data proving the antitumor activity of zinc and gold complexes in in vitro and in vivo experiments [13,14,15,16,17].

Zinc is one of the essential elements in humans and is therefore expected to be less toxic than platinum. It plays a critical role in a number of physiological processes and is important for the activity of thousands of enzymes and transcription factors. Zinc is involved in DNA synthesis and repair, plays a key role in monitoring cellular metabolism, and contributes to protection against oxidative damage. It is crucial for the normal development and functioning of the immune, endocrine, and nervous systems. Because of its importance, impairment of zinc homeostasis can lead to different diseases, including cancer [18,19]. Many studies have evidenced that Zn(II)-based complexes exhibit anticancer activity with low toxicity or have been proposed as photosensitizers in photodynamic therapy [13,14,15,16,17]. For example, a zinc complex derived from a cysteine-based Schiff base ligand (2-[(2-hydroxy-3-methoxy-benzylidene)-amino]-3-mercapto-propionic acid) showed significant apoptosis induction in both drug-sensitive CCRF-CEM and multidrug-resistant CEM/ADR5000 leukemia cell lines, while showing low toxicity toward normal human peripheral blood mononuclear cells (PBMCs) [20]. In another study, four heteroleptic Zn(II)-Schiff base complexes were investigated for their activity in triple-negative breast cancer (TNBC) cell lines. One of these complexes proved to be a promising agent for TNBC management, exhibiting IC_50_ values as low as to ~0.01 µM in some lines and reduced toxicity toward non-tumor lines. It inhibited colony formation and migration of TNBC cells and also sensitized them to doxorubicin and paclitaxel [21].

The biological activity of gold and its usefulness in medicine have been known since ancient times. The discovery of auranofin’s anticancer activity (an FDA-approved drug for the treatment of rheumatoid arthritis) has opened a new chapter in the exploration of gold complexes as anticancer drugs. There are a number of studies proving the antitumor activity of various gold complexes in in vitro and in vivo experiments.

Gold coordination compounds have been the subject of renewed interest in anticancer research, especially given the limitations of classical platinum-based drugs (e.g., cisplatin) [22,23,24,25,26,27]. Schiff base ligands provide a useful scaffold for Au(I)/(III) complexes, which can engage unique biological targets such as redox enzymes. Babgi et al. reported a series of Au(I) alkynyl complexes bearing phenolic Schiff base moieties that show anticancer activity against ovarian carcinoma (OVCAR-3) and non-small-cell lung cancer (HOP-62) cell lines. Notably, the para-hydroxy derivative had cytotoxicity comparable to cisplatin in OVCAR-3 [28]. A series of four gold(III) complexes bearing Schiff base ligands derived from the condensation of 1,2-bis(2-hydroxylphenyl)-1,2-diaminoethane and p-fluorobenzaldehyde was synthesized by Bian et al. All complexes exhibited significant notable antitumor activity against hepatocellular carcinoma cells [29]. Two gold(III) Schiff base complexes derived from 4-aminoantipyrine with ethylenediamine or benzaldehyde were reported by Melha et al. Both complexes exhibited higher cytotoxicity against HepG-2 cells than their corresponding free ligands, an effect attributed to chelation-enhanced lipophilicity and improved cellular uptake [30].

Although Schiff base complexes of Zn(II) or Au(I/III) have been investigated in a variety of tumor cells, relatively few studies have explicitly focused on glioblastoma. In one study, Alkiş et al. reported a novel Zn(II) complex with a Schiff base derived from the condensation of 4-aminopyrimidine-2(1H)-one with salicylaldehyde that exhibited anticancer potential against the human brain tumor cell lines T98G and U118 (both glioblastoma/astrocytoma types). The IC_50_ values were 282.47 µM (T98G) and 297.91 µM (U118), and 826.72 µM in healthy fibroblasts. It was shown that electroporation greatly enhanced the cytotoxic activity of the Zn(II) complex [31]. Gold coordination complexes, although often with non-Schiff base ligands, have demonstrated antitumor activity in glioblastoma models, frequently through the induction of mitochondrial dysfunction. For example, Greif et al. reported a rationally designed Au(I) complex ligated by N^N-bidentate ligands and supported by a N-heterocyclic ligand that modulates mitochondrial morphology to inhibit GBM in vitro and in vivo [32].

In a recent study conducted by our group on homo- and heterometallic complexes of Zn(II), {Zn(II)Au(I)}, and {Zn(II)Ag(I)} with pentadentate Schiff base ligands, we found that {Zn(II)Au(I)} heterometallic complexes exhibited the highest cytotoxic/antitumor activity against breast cancer (luminal type A and triple negative subtype) and cervical cancer cell lines [33]. Given these findings and considering the urgent need for effective therapies against glioblastoma, we aim to extend our investigations to glioblastoma cell lines. The present study aimed to evaluate the in vitro influence of six homometallic {Zn^II^_2_} and heterometallic {Zn^II^Au^I^} coordination compounds bearing Schiff base ligands on the viability, proliferation, and 2D/3D growth of 8MGBA and U251MG human glioblastoma cells. The investigated compounds were classified in two groups: Group I consisted of [Zn_2_(Ampy)(µ-OH)(H_2_O)_2_](ClO_4_)_2_ (**ZnAmpy**), [Zn_2_(Dmen)(µ-OH)(H_2_O)_2_](ClO_4_)_2_ (**ZnDmen**), ^1^_∞_[{Zn_2_(Ampy)(μ_3_-OH)}_2_(H_2_O){μ-[Au(CN)_2_]}](ClO_4_)_3_·THF·H_2_O (**ZnAmpyAu**), and [{Zn_2_(Dmen)(μ-OH)}_2_{μ-[Au(CN)_2_]}{[Au(CN)_2_]_2_}](ClO_4_)·H_2_O (**ZnDmenAu**); Group II consisted of ^1^_∞_[Zn(Salampy){μ-Au(CN)_2_}] (**ZnSalampyAu**) and ^1^_∞_[Zn(Saldmen)(μ-Au(CN)_2_}] (**ZnSaldmenAu**).

## 2. Results and Discussion

### 2.1. Synthesis

Two families of d^10^ homo- and heterometallic {Zn^II^Au^I^} complexes with Schiff bases derived from salicylaldehyde and 2,6-diformyl-*p*-cresol were investigated.

**Scheme 1:**
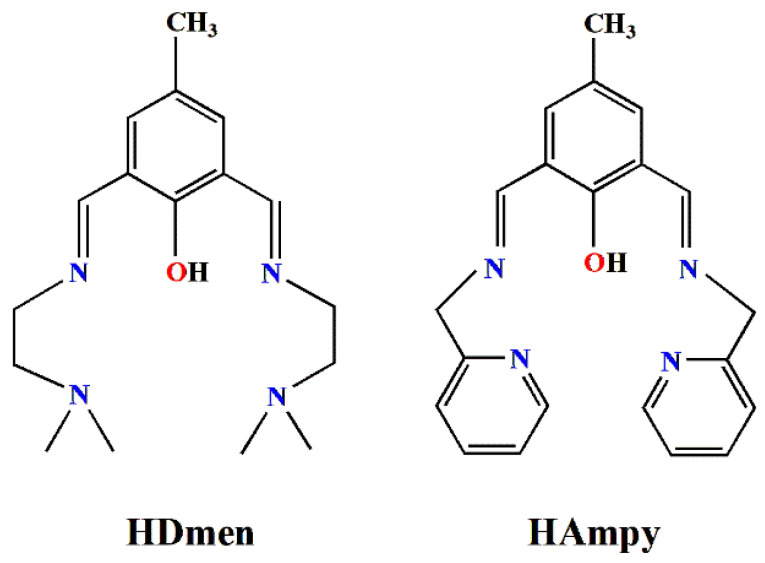
Schematic molecular structures of bicompartmental pentadentate ligands HDmen and HAmpy.

**Scheme 2:**
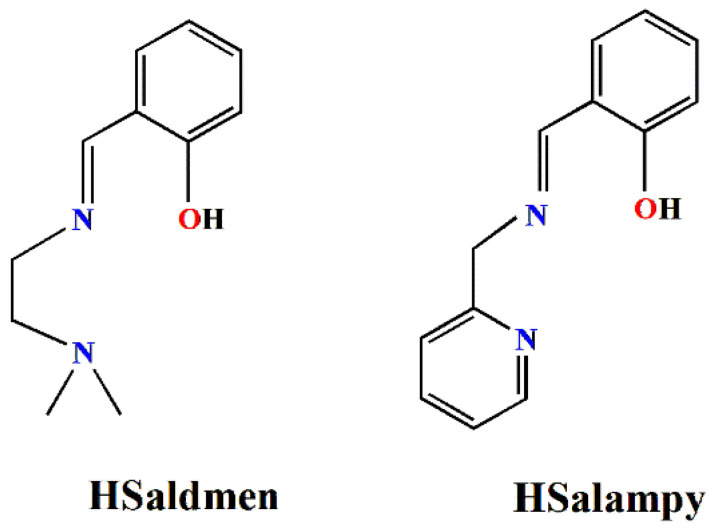
Schematic molecular structures of tridentate ligands HSaldmen and HSalampy.

The Schiff bases HDmen and HAmpy (Scheme 1) were synthesized by condensation of 2,6-diformyl-*p*-cresol with N,N-dimethylethylenediamine and 2-(aminomethyl)pyridine, respectively, while HSaldmen and HSalampy (Scheme 2) were obtained via condensation of salicylaldehyde with the same diamines.

The heterobimetallic complexes ^1^_∞_[Zn(Salampy){μ-Au(CN)_2_}] (**ZnSalampyAu**), [{Zn_2_(Dmen)(μ-OH)}_2_{μ-[Au(CN)_2_]}{[Au(CN)_2_]_2_}](ClO_4_)·H_2_O (**ZnDmenAu**), and ^1^_∞_[{Zn_2_(Ampy)(μ_3_-OH)}_2_(H_2_O){μ-[Au(CN)_2_]}](ClO_4_)_3_·THF·H_2_O (**ZnAmpyAu**) were prepared according to our previously reported methods [33,34,35].

**Scheme 3:**
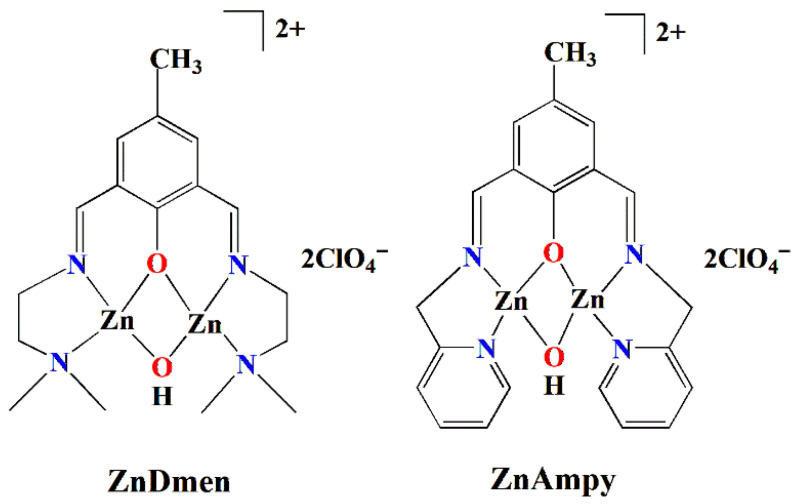
Schematic molecular structures of homobinuclear Zn(II) complexes (the water molecules coordinated at Zn(II) have been omitted for clarity).

The mononuclear precursors (in the case of **ZnSalampyAu** and **ZnSaldmenAu**) were synthesized in situ by reacting the corresponding Schiff bases with zinc(II) nitrate, as previously described [34]. The homobinuclear zinc(II) complexes **ZnDmen** and **ZnAmpy** (Scheme 3) were synthesized by reacting the HDmen and HAmpy Schiff bases with zinc(II) perchlorate, according to a method from the literature [33,36,37].

### 2.2. Structural Characterization

Since the synthesis and characterization of the **ZnDmen**, **ZnAmpy**, **ZnDmenAu**, **ZnAmpyAu**, and **ZnSalampyAu** complexes have been previously reported [33,34,35], only a brief description of their crystal structures is provided here.

**Figure 1:**
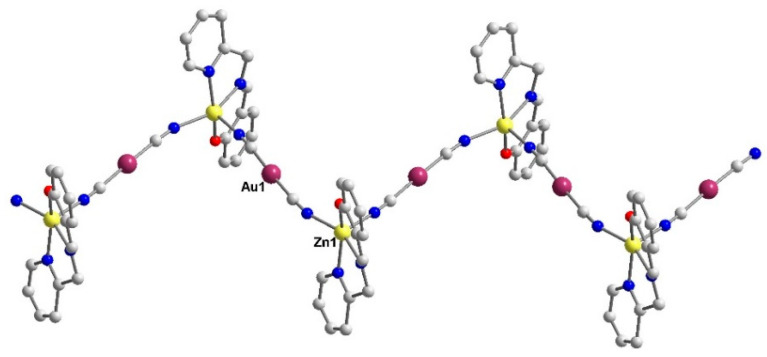
Perspective view of {ZnAu}_n_ zigzag chain in **ZnSalampyAu** (adapted from reference [34]).

Although the molar ratio between the starting compounds, complex cations, and complex anions was 1:1, the self-assembly process yielded distinct types of crystal structures. In **ZnSalampyAu** (Figure 1), polymeric zigzag chains are generated, composed of mononuclear Zn(II) nodes connected by [Au(CN)_2_]^−^ spacers [34].

**Figure 2:**
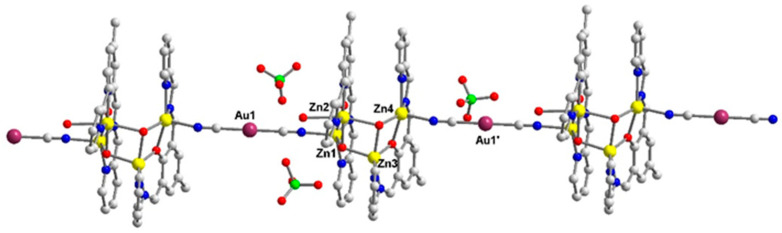
View of the coordination polymer in **ZnAmpyAu** (symmetry code ‘ = 1 + *x, y, z*] (adapted from reference [35]).

**ZnAmpyAu** forms a 1D coordination polymer (Figure 2) consisting of tetranuclear [Zn_4_]^4+^ nodes bridged by [Au(CN)_2_]^−^ anions; within the chain, the tetranuclear units are constructed from binuclear moieties joined by two hydroxo groups, which coordinate axially to a Zn(II) atom from a neighboring binuclear unit [35].

**Figure 3:**
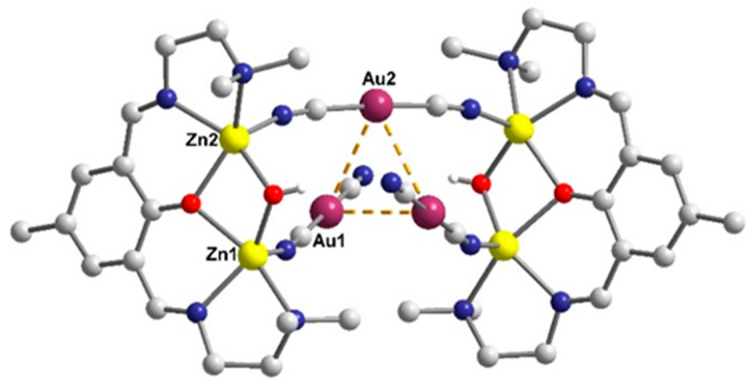
Perspective view of the heptanuclear complex in **ZnDmenAu** (the water molecules and the perchlorate anions were omitted for clarity) (adapted from reference [33]).

In contrast, a heptanuclear complex is observed in the structure of **ZnDmenAu** (Figure 3). The structure is made up of cationic heptanuclear entities [{Zn_2_(Dmen)(μ-OH)}_2_{μ-[Au(CN)_2_]}{[Au(CN)_2_]_2_}]^+^, perchlorate anions, and crystallization water molecules [33].

**Figure 4:**
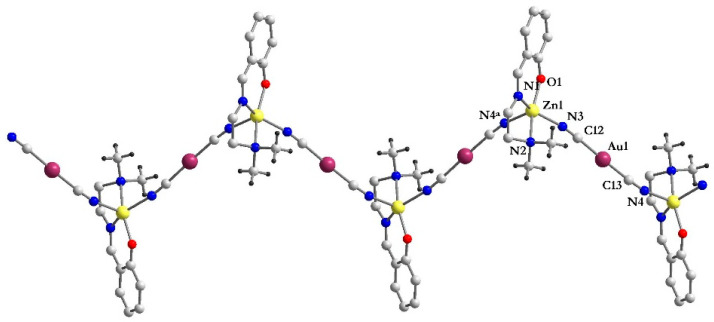
View of the polymeric zigzag chain in **ZnSaldmenAu** (symmetry code: a = 1 − *x*, −0.5 + *y*, 0.5 − *z*).

For the new synthesized compound **ZnSaldmenAu**, the crystallographic investigation reveals a one-dimensional coordination polymer with the formula ^1^_∞_[Zn(Saldmen)(μ-Au(CN)_2_}]. Its structure consists of neutral [Zn(Saldmen)(μ-Au(CN)_2_}] 1D polymeric zigzag chains (Figure 4). The Zn(II) centers are pentacoordinated with trigonal bipyramidal geometry. In this stereochemistry, the equatorial plane is formed by three nitrogen atoms and one imino from the Saldmen^−^ ligand and two from the two [Au(CN)_2_]^−^ bridges [Zn1-N1 = 2.039(7), Zn1-N3 = 2.093(7), Zn1-N4^a^ = 2.072(7) Å, symmetry code: a = 1 − *x*, −0.5 + *y*, 0.5 − *z*)]. The axial positions are occupied by a nitrogen and an oxygen atom from Saldmen^−^ [Zn1-N2 = 2.221(7) and Zn1-O1 = 1.954(6) Å]. The intra-node distance is Zn···Zn = 10.34 Å.

**Figure 5:**
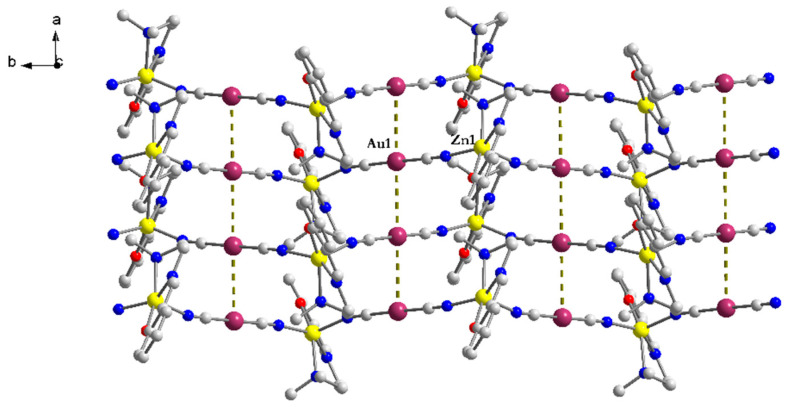
View of the packing diagram in **ZnSaldmenAu** through gold–gold interactions (Au···Au = 3.503 Å). Dotted green lines represent the Au···Au contacts.

At the supramolecular level, the gold(I) ions from neighboring chains are involved in aurophilic interactions, with an Au···Au separation of 3.503 Å, generating a 2D supramolecular structure (Figure 5 and Figure S1). The sum of the van der Waals radii of two Au(I) ions is 3.60 Å and is generally accepted as the maximum distance at which significant aurophilic interactions may occur [38,39]. Crystallographic data, structure refinement parameters, and selected bond distances referring to the stereochemistry of the metal ions are presented in Tables S1 and S2.

### 2.3. Spectral Characterization of ^1^_∞_[Zn(Saldmen){μ-Au(CN)_2_}] (ZnSaldmenAu)

#### 2.3.1. FT/IR and UV-Vis Spectra

The FT/IR spectrum of **ZnSaldmenAu** (Figure S2) exhibits a characteristic band at 2173 cm^−1^, corresponding to the cyanido bridges. The absence of any bands associated with the nitrate anion confirms its replacement by [Au(CN)_2_]^−^ units. A prominent band at 1653 cm^−1^, characteristic of the C=N stretching vibration, indicates the formation of the Schiff base ligand (Saldmen^−^). Additionally, the ν(C–O_phenoxido_) band appears in the 1240–1350 cm^−1^ range, consistent with coordination through the phenolic oxygen. The aliphatic –CH_3_ and –CH_2_– groups are observed at approximately 2998, 2968 cm^−1^ (ν_asym_ C–H), and 2888, 2844 cm^−1^ (ν_sym_ C–H), respectively. A broad absorption band in the 3400–3000 cm^−1^ region is attributed to water molecules and/or an extensive hydrogen bonding network.

**Figure 6:**
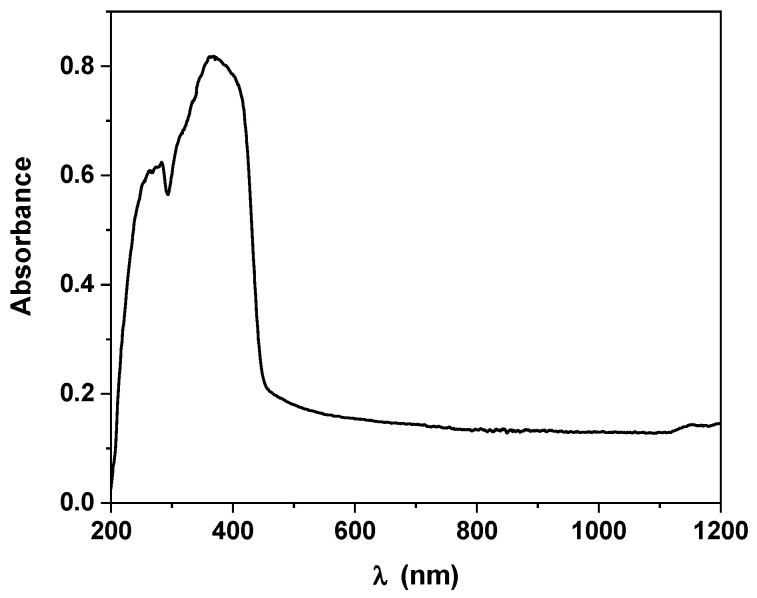
Electronic spectrum of **ZnSaldmenAu** (in solid state).

The electronic spectrum of **ZnSaldmenAu,** recorded in solid state, is presented in Figure 6. The spectrum displays absorption bands located in the 200–500 nm region. Usually, π–π* transitions of the organic ligand appear at higher energies, but in dicyanoaurate-containing polymers, strong metal-to-ligand charge transfer (MLCT, d–π*) transitions are expected within the same spectral region [35,40].

UV-Vis spectra of **ZnSaldmenAu** were also recorded in DMSO solution immediately after dissolution (t_0_) and after 24, 48, and 72 h to assess its stability in solution. The stability of the complex under conditions relevant to the biological assays was evaluated by comparing the UV–Vis spectra recorded at these time points (Figure S3). No significant spectral changes were observed over time, indicating that the complexes remain stable in DMSO under these experimental conditions.

#### 2.3.2. The ^1^H NMR Spectra

The ^1^H NMR spectrum of **ZnSaldmenAu** was recorded in DMSO-d6 (Figure S4a). The CH_3_ protons bonded to nitrogen in the ethylenediamine moiety appear at 2.31 ppm, while the CH_2_ protons of the same moiety are observed at 2.62 and 3.64 ppm. The azomethine protons appear as a singlet at 8.45 ppm, whereas the aromatic protons are observed at 6.47, 6.63, and 7. 18 ppm. The absence of a signal characteristic of the phenolic OH proton indicates deprotonation of this group and its involvement in coordination to the Zn(II) centers. The signal at 2.50 ppm is attributed to the free DMSO solvent, while that at 3.36 ppm is assigned to trace amounts of water in the solvent. To assess the stability of the complex in solution, time-dependent ^1^H NMR experiments were performed in DMSO over a period of 72 h (Figure S4b). The ^1^H NMR spectra demonstrate that **ZnSaldmenAu** remains stable over this time frame, as no changes in chemical shift positions were observed.

#### 2.3.3. Luminescence Properties

Zinc complexes with Schiff bases or aromatic heterocyclic ligands are known to exhibit noteworthy luminescent properties. Coordination of these ligands to the zinc(II) ion enhances the conformational rigidity of the ligand framework, thereby reducing energy loss through non-radiative thermal vibrations. Gold(I) complexes have garnered considerable interest not only in the field of medicine but also in the photophysics of metal-based systems. Both discrete or polymeric structures formed via aurophilic interactions are recognized as promising luminescent materials. In particular, aurophilic interactions play a significant role in the luminescent behavior of gold(I) complexes, contributing to emissions arising from dimers and to the luminescence of [Au(CN)_2_]^−^ in various environments. The emission energies of such compounds are highly sensitive to the distances between adjacent gold ions [41,42,43]. In this context, the photoluminescent behavior of the **ZnSaldmenAu** complex was investigated in solid state, as part of its physicochemical characterization. The corresponding emission spectrum, recorded at room temperature, is shown in Figure S5. Upon excitation at 370 nm, **ZnSaldmenAu** exhibits luminescence, with an emission maximum at λ_em_ = 450 nm. As observed for previously reported complexes, the luminescence in this case is most likely due to intraligand ^1^(π*–π) fluorescence.

### 2.4. Biological Evaluation

#### 2.4.1. Cytotoxic Assays

The cytotoxicity of the compounds (influence on cell viability and proliferation, cytopathological changes, and cell death) was studied in human 8MGBA and U251MG glioblastoma multiforme cells. The compounds were applied at a concentration range of 0.01–200 µg/mL for different treatment periods. Two groups of experiments were performed using methods with different mechanisms of action and cell targets (mitochondria, lysosomes, nuclei, DNA/RNA, etc.): short-term experiments (up to 72 h, MTT test, neutral red uptake cytotoxicity assay (NR), crystal violet staining technique (CV), double staining with acridine orange and propidium iodide, and Annexin V/FITC apoptosis/necrosis detection); and long-term experiments (up to 37 days using a 3D cell colony-forming method).

**Figure 7:**
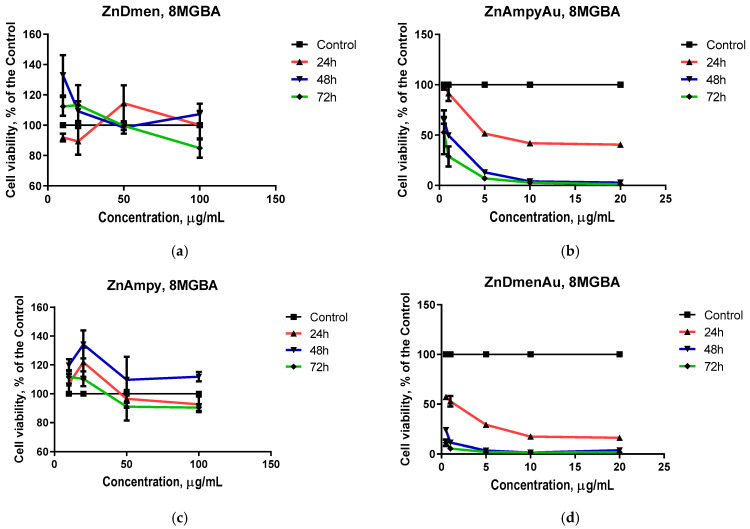
Cytotoxic activity of **ZnDmen** (**a**), **ZnAmpy** (**b**), **ZnAmpyAu** (**c**), and **ZnDmenAu** (**d**) in human 8MGBA cells. The cell viability and proliferation were determined by MTT test after 24, 48, and 72 h treatment periods.

**Figure 8:**
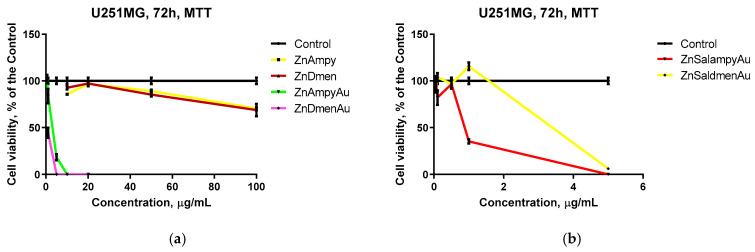
Cytotoxic activity of (**a**) **ZnAmpy**, **ZnDmen**, **ZnAmpyAu**, and **ZnDmenAu** and (**b**) **ZnSaldmenAu** and **ZnSalampyAu** in human U251MG cells. The cell viability and proliferation were determined by MTT test after a 72 h treatment period.

**Figure 9:**
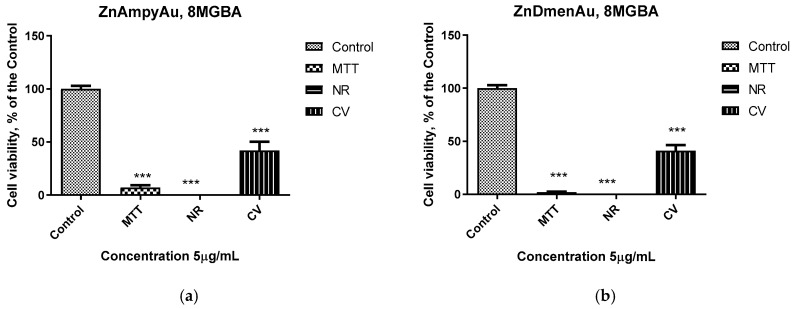
Experimental data obtained by MTT test (MTT), NR uptake cytotoxicity assay, and CV staining technique in human 8MGBA cells treated with 5 µg/mL **ZnAmpyAu** (**a**) and **ZnDmenAu** (**b**) after 72 h of incubation. (*p* < 0.001 = ***).

**Figure 10:**
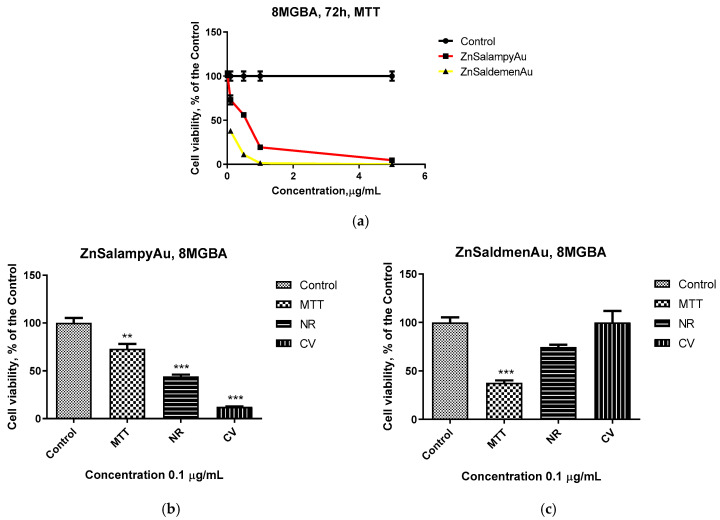
Cytotoxic activity of **ZnSaldmenAu** and **ZnSalampyAu** in human 8MGBA cells. Cell viability and proliferation were determined by MTT test after a 72 h treatment period (**a**). Experimental data obtained by MTT test, NR uptake cytotoxicity assay, and CV technique in human 8MGBA cells treated with 0.1 µg/mL **ZnSalampyAu** (**b**) and **ZnSaldmenAu** (**c**) after 72 h of incubation. (*p* < 0.01 = **; *p* < 0.001 = ***).

The results from the short-term experiments revealed that in both glioblastoma cell lines (8MGBA and U251MG) the compounds investigated decrease cell viability and proliferation in a time- and concentration-dependent manner. The {Zn^II^Au^I^} heterometallic complexes exhibit higher cytotoxic/antiproliferative activity compared to the Zn(II) homometallic complexes (Figure 7, Figure 8, Figure 9 and Figure 10 and Figures S6–S8). A good correlation was observed between data obtained by the three cytotoxicity assays MTT, NR, and CV (Figure 9 and Figure 10). The cytotoxic concentrations (CC_50_ and CC_90_, µg/mL) of the investigated compounds are summarized in Table 1, while those of the conventional antitumor agents vincristine, cisplatin, and oxaliplatin are shown in Table S3. Based on these values, the hierarchical orders of the investigated compounds and conventional antitumor agents were established (Table 2). The results showed that the compound with the highest cytotoxic activity in both permanent GBM cell lines from the I^st^ group is **ZnDmenAu**. From the II^nd^ group, the most effective compound in the 8MGBA cell line is **ZnSaldmenAu**, but in U251MG cells it is **ZnSalampyAu**. **ZnSalampyAu** was found to exhibit the highest cytotoxic activity against non-tumor Lep-3 cells.

It is worth noting that the activity of these metal complexes is superior to that of the conventional antitumor agents cisplatin, oxaliplatin, and vincristine. These results were confirmed by long-term experiments, where the compounds that fully inhibit the 3D colony-forming ability of human glioblastoma cell at the lowest concentrations in semi-solid medium are **ZnDmenAu** (8MGBA, U251MG), **ZnSaldmenAu** (8MGBA), and **ZnSalampyAu** (U251MG). 

#### 2.4.2. Double Staining with Acridine Orange and Propidium Iodide (AO/PI)

**Figure 11:**
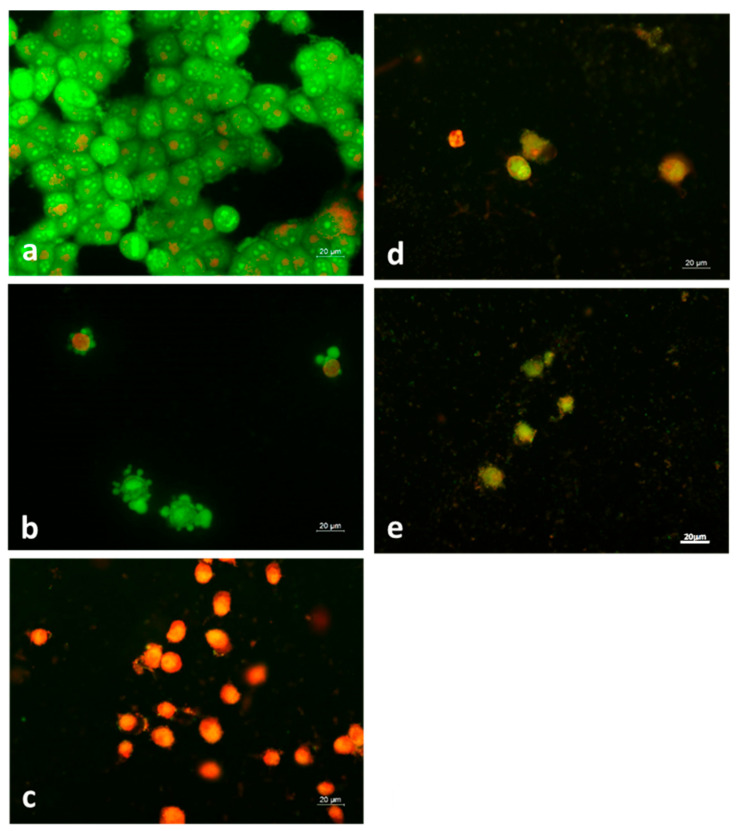
Cytopathological changes in 8MGBA cells. Cells pre-cultured for 24 h in culture medium ((**a**) Control) and cells treated with {Zn^II^Au^I^} complexes at 2.5 µg/mL concentration ((**b**) **ZnAmpyAu**; (**c**) **ZnDmenAu**; (**d**) **ZnSalampyAu**; (**e**) **ZnSaldmenAu**) for 24 h. Double staining with AO/PI. Fluorescence microscope (Leika DM 500B, Wetzlar, Germany) (magnification 40×). Bar = 20 µm.

**Figure 12:**
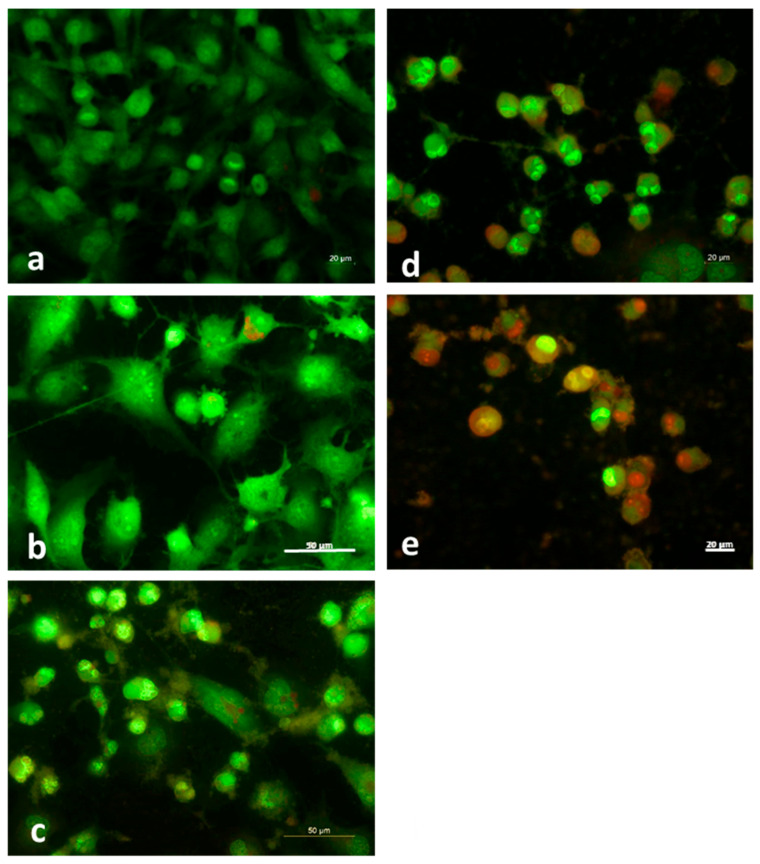
Cytopathological changes in U251MG cells. Cells pre-cultured for 24 h in culture medium ((**a**) Control) and cells treated with {Zn^II^Au^I^} complexes in 1 µg/mL concentration ((**b**) **ZnAmpyAu**; (**c**) **ZnDmenAu**; (**d**) **ZnSalampyAu**; (**e**) **ZnSaldmenAu**) for 24 h. Double staining with AO/PI. Fluorescence microscope (Leika DM 500B, Wetzlar, Germany) (magnification 40×). Bar = 20 µm.

Double staining with AO/PI did not show any morphological changes in untreated cells from both glioblastoma cell lines (8MGBA and U251MG); bright green intact cells and the presence of multiple mitoses were found. In contrast, cells cultured in the presence of the tested metal complexes revealed signs typical of the early and late phases of apoptosis. These were cells with fragmented nuclei, chromatin condensation, and membrane blebbing. The presence of an orange color due to the binding of PI to denatured DNA indicates loss of cell membrane integrity and dead cells (Figure 11 and Figure 12).

#### 2.4.3. Apoptosis Detection by Annexin V-FITC Staining Method

**Figure 13:**
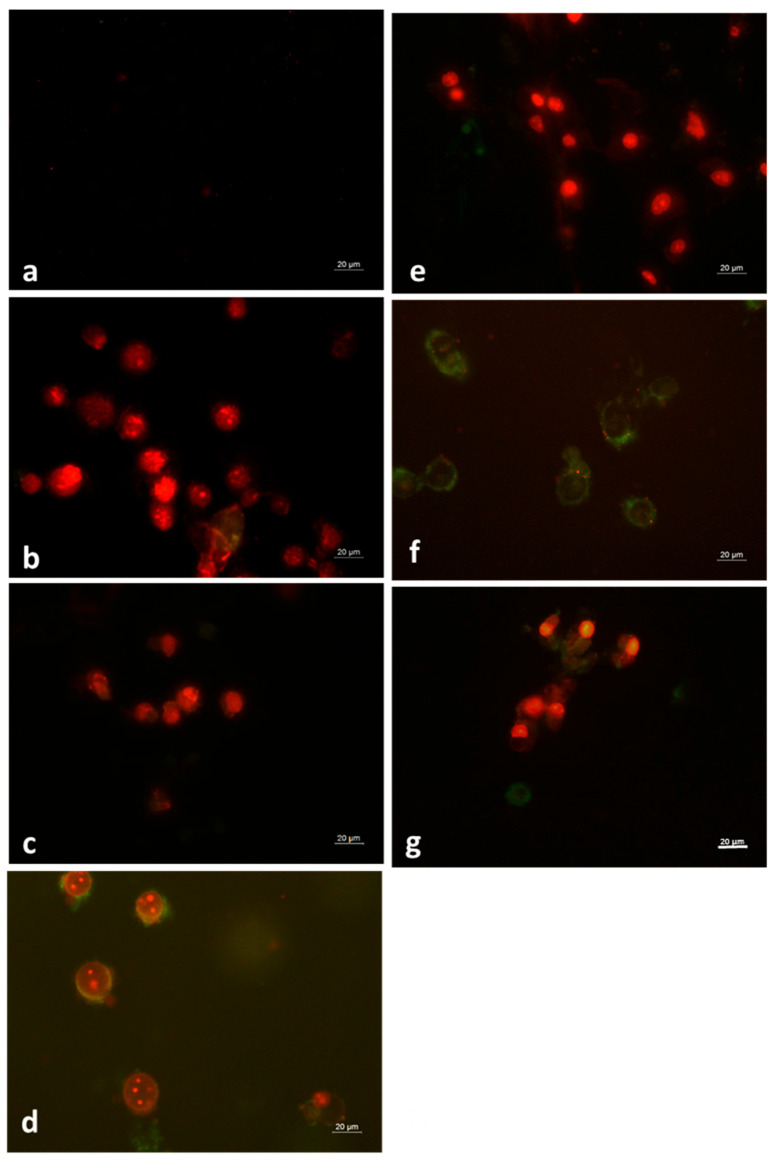
Detection of apoptosis in 8MGBA cells by Annexin-V-FITC and PI staining. Cells pre-cultured for 24 h in culture medium ((**a**) Control) and treated with different concentrations of {Zn^II^Au^I^} complexes ((**b**) **ZnAmpyAu** 1 µg/mL; (**c**) **ZnDmenAu** 0.5 µg/mL; (**d**) **ZnSalampyAu** 0.5 µg/mL; (**e**) **ZnSalampyAu** 1 µg/mL; (**f**) **ZnSaldmenAu** 0.5 µg/mL, and (**g**) **ZnSaldmenAu** 1 µg/mL) for 24 h. After the incubation period, cells were stained with Annexin V-FITC and PI and analyzed by fluorescence microscopy (magnification 40×). Bar = 20 µm.

**Figure 14:**
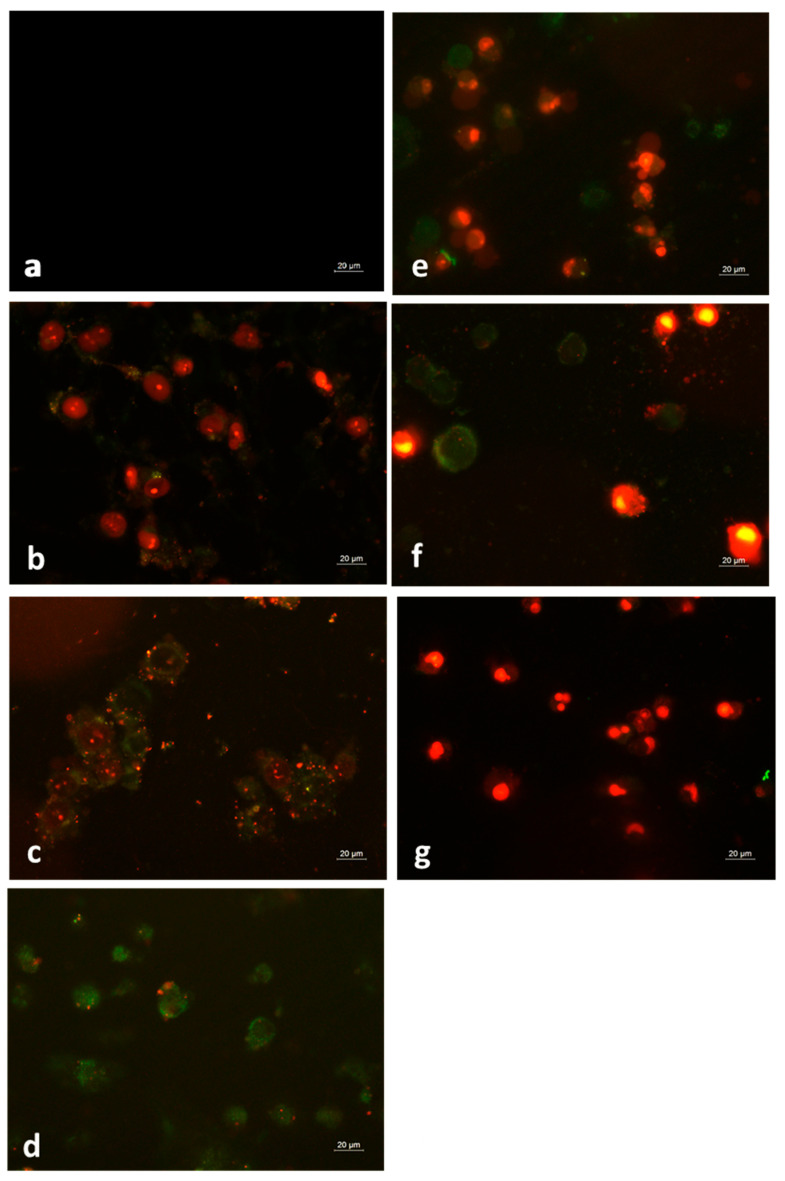
Detection of apoptosis in U251MG cells by Annexin-V-FITC and PI staining. Cells pre-cultured for 24 h in culture medium ((**a**) Control) and treated with different concentrations of {Zn^II^Au^I^} complexes ((**b**) **ZnAmpyAu** 1 µg/mL; (**c**) **ZnDmenAu** 0.5 µg/mL; (**d**) **ZnSalampyAu** 0.5 µg/mL; (**e**) **ZnSalampyAu** 1 µg/mL; (**f**) **ZnSaldmenAu** 0.5 µg/mL, and (**g**) **ZnSaldmenAu** 1 µg/mL) for 24 h. After the incubation period, cells were stained with Annexin V-FITC and PI and analyzed by fluorescence microscopy (magnification 40×). Bar = 20 µm.

The experiments conducted confirmed the ability of the studied compounds to induce apoptosis in treated human glioblastoma cells. In the early stages of apoptosis, Annexin V binds to phosphatidylserine (PS) that is exposed on the outer membrane, but PI cannot penetrate into the cells (green fluorescence). In the late stages of apoptosis, Annexin V binds to PS exposed on the outer membrane but the integrity of the plasma membrane is lost and PI enters the cell and intercalates with double-stranded DNA (red fluorescence) (Figure 13 and Figure 14).

#### 2.4.4. 3D Cell Colony Formation

In long-term experiments, it was found that the compounds investigated inhibit 3D cell colony formation in a semi-solid medium in both glioblastoma cell lines (8MGBA and U251MG). It is striking that a complete absence of colonies/cells was observed even at the lowest colony inhibitory concentrations (CIC) of **ZnDmenAu** and **ZnSaldmenAu** (0.5 µg/mL), with the effect persisting over time (Table 3).

Compared to the Zn(II) homometallic complexes, **ZnAmpy** and **ZnDmen,** which showed no inhibitory activity up to 100 μg/mL, the presence of Au(I) in the {Zn^II^Au^I^} complexes substantially enhances their biological performance. This is consistent across all {Zn^II^Au^I^} complexes but most pronounced in those bearing the Dmen^−^ and Saldmen^−^ ligands. The data indicate a synergistic or additive effect arising from the presence of Au(I) in the system. The hierarchical orders summarized in Table 4 clearly reflect this trend. In both glioblastoma cell lines, **ZnDmenAu** and **ZnSaldmenAu** emerge as the most potent inhibitors, followed closely by **ZnSalampyAu**, while **ZnAmpyAu** shows moderate activity. By contrast, **ZnAmpy** and **ZnDmen** consistently exhibit minimal effects. Notably, the ranking pattern is conserved across the two glioblastoma lines, suggesting that the enhanced activity of the {Zn^II^Au^I^} complexes is not limited to a specific cell type. These findings indicate **ZnDmenAu** and **ZnSaldmenAu** as the most promising candidates within the series, displaying potent and durable inhibition of 3D cell colony formation.

Treatment of glioblastoma multiforme is still a big challenge in clinical practice due to its high degree of malignancy, high heterogeneity and strong invasiveness, as well as the limited access of antitumor agents to the tumor. Last but not least are the side effects of the conventional antitumor agents. Modern methods of treatment include a combined approach involving chemotherapy (Temozolomide), radiotherapy, and surgery. After this treatment, the survival rate of those diagnosed with this disease increases by only 2.5%. Searching for new ways to overcome the blood–brain barrier, as well as new agents that induce a high antitumor effect with less side and toxic effects, continues.

The results obtained by us open the possibility that a new antitumor agent can be found. {Zn^II^Au^I^} complexes with Schiff base ligands showed a promising antitumor activity in the experimental models used in our experiments, i.e., the permanent human GBM cell lines 8MGBA and U251MG. Special attention should be paid to **ZnDmenAu**. It was found to be the most active compound compared to the others investigated. This result was confirmed across all the methods employed, i.e., in both short-term and long-term experiments. We compared the compounds investigated to some antitumor agents used in clinical practice against tumor diseases, including GBM (cisplatin, oxaliplatin, and vincristine). The results demonstrated that in most cases, {Zn^II^Au^I^} heterometallic complexes exhibit higher cytotoxic/antiproliferative activity compared to cisplatin, vincristine, and oxaliplatin (Table 2, Figures S9 and S10). **ZnDmenAu** deserves special attention, as it showed higher antitumor activity than these conventional agents.

The cell lines used show different sensitivities to the cytotoxic effect of the compounds investigated. In most cases, 8MGBA cells show higher sensitivity compared to U251MG cells (Figures S7 and S8). The high sensitivity of non-tumor embryonic cells from the Lep-3 line is also impressive (Table 2). The observed cell-specific response may be due to various factors, including the unique profile of each tumor/tumor cell line. Although derived from the same tumor type—glioblastoma multiforme—the 8MGBA and U251MG cell lines have individual characteristics (high/low expression of GFAP, EGFR, vimentin, etc.), which affect their biological behavior. The high sensitivity of embryonic cells compared to tumor cells is known—in the course of tumor progression, cell subpopulations with increased survival and adaptation abilities are selected.

More investigations (additional methods and experimental models) are needed to reveal the mechanisms of action of these compounds. A potential antitumor drug has a long way to go from the laboratory to clinical practice. For the first time, {Zn^II^Au^I^} complexes with Schiff base ligands were evaluated regarding their potential antitumor activity in human GBM cells.

## 3. Materials and Methods

### 3.1. Materials

All chemicals used in this study were of reagent grade, obtained from commercial suppliers, and used without further purification. Salicylaldehyde > 99%, N,N-dimethylethylenediamine > 98%, 2-(aminomethyl)pyridine > 99%, zinc(II) nitrate tetrahydrate > 99% (Zn(NO_3_)_2_·4H_2_O), zinc(II) perchlorate hexahydrate (Zn(ClO_4_)_2_·6H_2_O), potassium dicyanoaurate(I) 98% (K[Au(CN)_2_]), lithium hydroxide > 98% (LiOH), triethylamine > 99%, and methanol > 99.9% (MeOH) were purchased from Merck Millipore. 2,6-Diformyl-*p*-cresol was prepared according to Okawa et al. [44].

The organic pro-ligands HSaldmen, HSalampy, HDmen, and HAmpy were synthesized in situ by the condensation reaction of salicylaldehyde and 2,6-diformyl-*p*-cresol with N,N-dimethyl-ethylenediamine and 2-(aminomethyl)pyridine, respectively.

The heterobimetallic complexes ^1^_∞_[Zn(Salampy){μ-Au(CN)_2_}] (**ZnSalampyAu**), [{Zn_2_(Dmen)(μ-OH)}_2_{μ-[Au(CN)_2_]}{[Au(CN)_2_]_2_}](ClO_4_)·H_2_O (**ZnDmenAu**), and ^1^_∞_[{Zn_2_(Ampy)(μ_3_-OH)}_2_(H_2_O){μ-[Au(CN)_2_]}](ClO_4_)_3_·THF·H_2_O (**ZnAmpyAu**) were prepared according to our previously reported methods [33,34,35].

Dulbecco’s modified Eagle’s medium (D-MEM) and fetal bovine serum were obtained from Gibco-Invitrogen (UK). Purified agarose, thiazolyl blue tetrazolium bromide (MTT), acridine orange (3,6-Acridinediamine, N,N,N’,N’-tetramethyl-, monohydrochloride), propidium iodide, and Ni(CH_3_COO)_2_·4H_2_O were purchased from Sigma-Aldrich Chemie GmbH (Schnelldorf, Germany). Dimethylsulfoxide (DMSO) and trypsin were obtained from AppliChem (Darmstadt, Germany). The antibiotics (penicillin and streptomycin) were from Lonza (Belgium). All the other chemicals, of the highest purity commercially available, were purchased from local agents and distributors. All sterile plasticware and syringe filters were from Orange Scientific (Braine-l’Alleud, Belgium). The Annexin V Apoptosis Detection kit was purchased from Santa Cruz Biotechnology, Inc. (Heidelberg, Germany).

### 3.2. Synthesis of ^1^_∞_[Zn(Saldmen){μ-Au(CN)_2_}] (ZnSaldmenAu)

Methanolic solutions containing stoichiometric amounts of salicylaldehyde (0.5 mmol in 15 mL) and N,N-dimethylethylenediamine (0.5 mmol in 10 mL) were mixed and stirred continuously at 50 °C for 30 min. Subsequently, stoichiometric amounts of triethylamine (0.5 mmol in 10 mL MeOH) and Zn(NO_3_)_2_·4H_2_O (0.5 mmol in 15 mL MeOH) were added to the reaction mixture, which was then stirred for an additional 30 min. The final yellow solution was carefully layered over an aqueous solution (5 mL) of K[Au(CN)_2_] (0.5 mmol). This compound was obtained as a yellow crystalline precipitate. Slow evaporation at room temperature of the light-yellow filtrate led to pale yellow crystals after several days. The crystallinity and phase purity of **ZnSaldmenAu** was confirmed by powder X-ray diffraction (PXRD), which closely matched the simulated diffractograms from single-crystal X-ray data (Figure S11). Elemental chemical analysis (%): C, 30.05; H, 2.97; N, 11.07 (calcd.); C, 29.52, H, 3.60, N, 10.81 (found). IR data (KBr, cm^−1^): 3384m, 2998w, 2967w, 2888m, 2844w, 2796w, 2173vs, 1635vs, 1598m, 1538m, 1469vs, 1448s, 1402m, 1344m, 1284m, 1249w, 1191m, 1151m, 1128w, 1075w, 1027w, 952w, 902m, 854ws, 794w, 761s, 624w, 611w, 528w, 457w.

All the synthesized complexes are air-stable, freely soluble in DMSO and DMF, and slightly soluble in water.

*Caution!* Perchlorate salts are potentially explosive and should be handled in small quantities.

### 3.3. Physicochemical Characterization Methods

Elemental chemical analysis of C, H, and N were performed on a EuroEA Elemental Analyzer (HEKAtech GmbH, Wegberg, Germany). FT/IR spectra were recorded as KBr pellets on a JASCO FTIR 4100 spectrophotometer (JASCO International Co., Ltd., Tokyo, Japan) in the range of 4000–400 cm^−1^. Electronic spectra were recorded in solid state, using the diffuse reflectance technique, with a JASCO V-670 spectrophotometer (JASCO International Co., Ltd., Tokyo, Japan). The photoluminescence measurements were carried out at room temperature using a JASCO FP 6500 spectrofluorometer (JASCO International Co., Ltd., Tokyo, Japan). The ^1^H NMR spectra were recorded on a Bruker Avance III Ultrashield Plus 500 MHz spectrometer (Bruker, Karlsruhe, Germany) operating at 11.74 T, corresponding to the resonance frequency of 500.13 MHz for the ^1^H nucleus, equipped with a direct detection four nuclei probe head and field gradients on the *z* axis.

The single-crystal X-ray diffraction measurements were performed on a Rigaku XtaLAB Synergy, single source at offset/far, HyPix diffractometers ((Rigaku, Tokyo, Japan) operating with a graphite-monochromated Mo Kα radiation source (λ = 0.71073 Å). The structure was solved by direct methods and refined using full-matrix least squares techniques based on F^2^. The non-H atoms were refined with anisotropic displacement parameters. Hydrogen atoms were refined with a riding model. Calculations were performed using the SHELXL-2015/2018 crystallographic software package [45,46]. A summary of the crystallographic data and the structure refinement parameters for crystal **ZnSaldmenAu** are given in Table S1. CCDC reference number: 2517579.

Powder X-ray diffraction (PXRD) patterns were collected using a Rigaku Ultima IV diffractometer (Rigaku, Tokyo, Japan) with CuKα radiation (λ = 1.5406 Å) in the 2θ range of 3–50°, at a scanning speed of 2° min^−1^ and a step size of 0.02°, operating at 40 kV and 30 mA.

### 3.4. Cell Cultures and Cultivation

Two permanent cell lines established from human glioblastoma multiforme were used as model systems in our investigations—8 MGBA and U251MG. The permanent cell line Lep-3 (human non-tumor embryonal fibroblastoid cells) was used in some of the experiments for comparative purposes. The cells were grown as monolayer cultures in D-MEM medium, supplemented with 5–10% fetal bovine serum, 100 U/mL penicillin, and 100 g/mL streptomycin. The cultures were maintained at 37 °C in a humidified CO_2_ incubator Hepa class 100 (ThermoFisher Scientific, Darmstadt, Germany). For routine passages, adherent cells were detached using a mixture of 0.05% trypsin and 0.02% ethylenediamine tetraacetic acid (EDTA).

### 3.5. Cytotoxicity Assays

Cytotoxicity assays were performed as previously described [33]. The cells were seeded in 96-well flat-bottomed microplates at a concentration of 1 × 10^4^ cells/well. After the cells were grown for 24 h to a subconfluent state (~ 60–70%), the culture medium was removed and replaced by media modified with different concentrations (0.1, 0.5, 1, 5, 10, 20, 50, 100 µg/mL) of the compounds tested. Each concentration was applied into 4–8 wells. Samples of cells grown in non-modified medium served as controls (culture medium control). The effect of the compounds on cell viability and proliferation was examined by MTT test [47] (MTT, 24, 48, 72 h), neutral red uptake cytotoxicity assay [48] (NR, 72h), and the crystal violet staining technique [49] (CV, 72h). Concentration–response curves were prepared and the effective cytotoxic concentration (CC) of the compounds, CC50 (μM) and CC90 (μM), causing, respectively, 50% and 90% reduction of cell viability compared to the control were estimated from these curves. All data points represent an average of three independent assays [33].

The commercially available antitumor agents cisplatin, oxaliplatin, and vincristine, were included as positive controls in our investigations. They were dissolved according to the manufacturer’s instructions.

### 3.6. Double Staining with Acridine Orange (AO) and Propidium Iodide (PI)

The cells were seeded on sterile coverslips in 6-well plates at a density of 3–3.5 × 10^5^ cells/well and cultivated for 24–72 h in the presence of the compounds tested. A culture medium control was also prepared. After incubation was completed, the coverslips were removed and washed with phosphate-buffered saline (PBS), pH 7.2–7.4, for 2 min. The cells were stained using a mixture (1:1/vol.:vol.) of fluorescent dyes containing AO (10 µg/mL in PBS) and PI (10 µg/mL in bidistilled water) [50]. The cells were observed using a fluorescence microscope (LeikaDM 500B, Wetzlar, Germany) no later than 30 min after staining to avoid attenuation of the fluorescence signal.

### 3.7. Cell Death Identification

The exposure of phosphatidylserine (PS) residues on the cell membrane surface represents an early event in the apoptotic cascade and serves as a reliable marker for its detection and quantification. During apoptosis, phosphatidylserine is translocated from the cytosolic side of the plasma membrane (where it is normally located) to its outer surface, facilitating its detection. In our experiments, an apoptosis kit (Annexin V apoptosis detection kit (sc-4252 AK), Immuno Cruz) was used. Cells were cultured on coverslips placed in 6-well plates at a density of 3.0−3.5 × 10^5^ cells per well. At 24 h, the culture medium was aspirated and replaced with medium containing different concentrations of the tested compounds. After an incubation period of 72 h, the coverslips were washed once with 1 x PBS (2.68 mM KCl, 1.47 mM KH_2_PO_4_, 1.37 mM NaCl, 8 mM Na_2_HPO_4_, pH 7), after which buffer (1× Assay Buffer: solution of 1part 10x Assay Buffer in 9 parts H_2_O) was added to each well. Amounts of 5 μL Apoptosis Detection reagent/Annexin V-FITC and 10 μL propidium iodide were added to each well according to the manufacturer’s protocol. The samples were incubated at room temperature for 15 min in the dark. Finally, the cells were washed again with 1x PBS, pH 7. The presence/absence of apoptosis was observed using a fluorescence microscope at the wavelength (λ = 488 nm) recommended by the manufacturer (LeikaDM 500B, Wetzlar, Germany).

### 3.8. 3D Cell Colony-Forming Assay

The investigations were performed as earlier described [33]. Tumor cells (approximately 10^3^ cells/well) suspended in 0.45% purified agarose in 2× D-MEM medium containing different concentrations of the compounds examined (ranging from 0.01 to 200 µg/mL) were layered in 24-well microplates. The presence/absence of colonies was registered using an inverted light microscope (Carl Zeiss, Jena, Germany) for a period longer than two weeks. The colony inhibitory concentration (CIC) at which no 3D cell colony formation was observed in the well (only single cells or no cells were found) was determined.

### 3.9. Statistical Analysis

The data are presented as mean ± standard error of the mean. Statistical differences between control and treated groups were assessed using one-way analysis of variance (ANOVA) followed by Dunnett’s post hoc test. The results from FACS analysis were quantified with FlowJo_V10 software.

## 4. Conclusions

In this study, we investigated six homo- and heterometallic {Zn^II^_2_} and {Zn^II^Au^I^} complexes bearing Schiff base ligands resulting from the condensation of 2,6-diformyl-*p*-cresol and salicylaldehyde, respectively, with N,N-dimethylethylenediamine and 2-(aminomethyl)pyridine, for their cytotoxic and antiproliferative effects against 8MGBA and U251MG human glioblastoma multiforme cell lines. The new compound, **ZnSaldmenAu,** reported here for the first time, was thoroughly characterized using a combination of structural and spectroscopic techniques. Its stability in DMSO solution, relevant for the biological studies, was confirmed over a period of 72h by UV-Vis and ^1^H NMR spectroscopy. Across multiple complementary assays, all compounds exhibited a promising ability to decrease the viability and 2D/3D cell growth of tumor cells, highlighting their potential as anticancer agents. Notably, the heterometallic coordination compounds containing both Zn(II) and Au(I) centers showed enhanced biological activity compared to the Zn(II) homometallic complexes, supporting their relevance as promising candidates in glioblastoma drug development. The tested compounds affected the two cell lines differently, with 8MGBA cells generally showing greater sensitivity than U251MG cells. **ZnDmenAu** emerged as the most active compound among those examined. Its strong cytotoxic effect was confirmed across all applied methods in both short-term and long-term experiments. When compared with clinically used antitumor agents—cisplatin, oxaliplatin, and vincristine—the {Zn^II^Au^I^} heterometallic complexes generally demonstrated superior cytotoxic activity against glioblastoma cells. Although further mechanistic studies and in vivo validation are required, these results provide a strong experimental foundation for the continued investigation of these compounds as potential candidates for glioblastoma therapy.

## Figures and Tables

**Table 1:** Cytotoxic concentrations, CC_50_ * and CC_90_ ** (in parentheses) (µM), of the investigated compounds in cultured human glioblastoma (8MGBA and U251MG) and non-tumor (Lep-3) cells.

Compound	Cytotoxic Concentration CC_50_ and (CC_90_) (µM)					
	8MGBA				U251MG	Lep-3
	Method	Incubation Period (h)				
		24	48	72	72	72
**ZnSalampyAu**	MTT	n.da.	n.da.	1.10 ± 1.2 * (6.72) ± 2.4 **	1.5 ± 0.9 (7.1 ± 2.3)	0.57 ± 1.8 (0.89 ± 1.6)
**ZnSaldmenAu**	MTT	n.da.	n.da.	0.17 ± 0.9 (0.57 ± 1.4)	6.74 ± 2.1 (9.63 ± 1.2)	1.03 ± 1.4 (0.93 ± 2.2)
**ZnDmen**	MTT	-	-	-	-	41.7 ± 2.4 (71.01 ± 2.6)
**ZnAmpy**	MTT	-	-	-	-	24.8 ± 2.3 (65.4 ± 1.8)
**ZnAmpyAu**	MTT	3.58 ± 1.1 (-)	0.57 ± 1.7 (4.04 ± 3.3)	0.33 ± 2.4 (2.72 ± 2.2)	18.7 ± 2.6 (6.09 ± 2.0)	0.6 ± 1.7 (2.62 ± 2.5)
**ZnDmenAu**	MTT	0.83 ± 0.9 (-)	n.d.	n.d.	n.d.	0.73 ± 1.4 (2.55 ± 2.1)

“-”—the concentration cannot be calculated because the viability of the cells is >50% or >10%; “n.d.”—not determined because the viability of the cells is <50% or <10%; “n.da.”—no data.

**Table 2:** Hierarchical orders of the investigated compounds and conventional antitumor agents according to their CC_50_ * and CC_90_ ** in human 8MGBA, U251MG, and Lep-3 cells, as determined by MTT test after 72 h of treatment.

Cell Line	Treatment Period (h)	Hierarchical Order
8MGBA	72	* ZnDmenAu ≥ ZnSaldmenAu > ZnAmpyAu > ZnSalampyAu > CisPt > (Vincristine; OxPt; ZnAmpy; ZnDmen) ** (ZnDmenAu > ZnSaldmenAu > ZnAmpyAu > ZnSalampyAu > Vincristine ≥ CisPt > OxPt ≥ (ZnAmpy; ZnDmen)
U251MG	72	ZnDmenAu > ZnSalampyAu > CisPt > ZnSaldmenAu > ZnAmpyAu > OxPt > (Vincristine; ZnAmpy; ZnDmen) (ZnDmenAu > ZnAmpyAu > ZnSalampyAu > ZnSaldmenAu > CisPt > OxPt > (Vincristine; ZnAmpy; ZnDmen))
Lep-3	72	ZnSaldmenAu > ZnSalampyAu > ZnDmenAu > ZnAmpyAu = CisPt > OxPt > ZnAmpy > ZnDmen (ZnSaldmenAu > ZnSalampyAu > ZnDmenAu > ZnAmpyAu > ZnAmpy > CisPt > ZnDmen > OxPt)

Each hierarchical order begins with the most active compound.

**Table 3:** 3D Colony inhibitory concentration (CIC, µg/mL).

Compound	8MGBA		U251MG	
	Observation Period, Days			
	26	37	21	28
**ZnAmpy**	≥100	≥100	≥100	≥100
**ZnDmen**	≥100	≥100	≥100	≥100
**ZnAmpyAu**	≥20	≥5	≥5	≥5
**ZnDmenAu**	≥10	≥0.5	≥5	≥1
	**16**	**23**	**14**	**24**
**ZnSalampyAu**	≥1	≥1	≥5	≥1
**ZnSaldmenAu**	≥1	≥0.5	≥5	≥1

**Table 4:** Hierarchical orders of {Zn^II^Au^I^} complexes according to their CIC (µg/mL) in human 8MGBA and U251MG glioblastoma cells.

Cell Line	Hierarchical Order
8MGBA	ZnDmenAu = ZnSaldmenAu > ZnSalampyAu > ZnAmpyAu > ZnAmpy = ZnDmen
U251MG	ZnDmenAu = ZnSaldmenAu = ZnSalampyAu > ZnAmpyAu > ZnAmpy = ZnDmen

Each hierarchical order begins with the most active compound.

## Data Availability

The original contributions presented in this study are included in the article. Further inquiries can be directed to the corresponding authors.

## Supplementary Materials

The following supporting information can be downloaded at: https://www.mdpi.com/article/10.3390/molecules31010173/s1, Figure S1: View of packing diagram of **ZnSaldmenAu** along the crystallographic *a* axis; Figure S2. The FT/IR spectrum of **ZnSaldmenAu**; Figure S3. UV-Vis spectra of **ZnSaldmenAu** in DMSO solution at t_0_ and after 24, 48, and 72 h; Figure S4. ^1^H NMR (DMSO-d6) spectra of **ZnSaldmenAu**: (a) initial spectrum and (b) recorded at t_0_ and after 24, 48, and 72 h; Figure S5. Solid-state emission spectrum of **ZnSaldmenAu** at room temperature (λ_exc_ = 370 nm); Figure S6. Cytotoxic activity of **ZnAmpy**, **ZnDmen**, **ZnAmpyAu**, and **ZnDmenAu** in human 8MGBA cells. The cell viability and proliferation were determined by MTT test after 24 (a), 48 (b), and 72 h (c) treatment periods; Figure S7. Cytotoxic activity of **ZnAmpy** (a), **ZnDmen** (b), **ZnAmpyAu** (c), and **ZnDmenAu** (d) in human 8MGBA and U251MG cells. Cell viability and proliferation were determined by MTT test after a 72 h treatment period; Figure S8. Cytotoxic activity of **ZnSalampyAu** (a) and **ZnSaldmenAu** (b) in human 8MGBA and U251MG glioblastoma cells. The cell viability and proliferation were determined by MTT test after a 72 h treatment period; Figure S9. Glioblastoma multiforme cells (8MGBA permanent cell line) treated with cisplatin (a), oxaliplatin (b), and vincristine (c) for 24, 48, and 72 h. Cell viability was evaluated by MTT test; Figure S10. Glioblastoma multiforme cells (U251MG permanent cell line) treated with cisplatin (a), oxaliplatin (b), and vincristine (c) for 24, 48, and 72 h. Cell viability was evaluated by MTT test; Figure S11. X-ray powder diffractograms of **ZnSaldmenAu** simulated from the SC-XRD data (red line) and experimental PXRD pattern (black line); Table S1. Crystallographic details of data collection and structure refinement parameters for compound **ZnSaldmenAu**; Table S2. Selected geometric parameters: bonds (Å) and angles (°) in compound **ZnSaldmenAu**; Table S3. Cytotoxic activity CC_50_ * and CC_90_ ** (in parentheses) (µM) of conventional antitumor agents vincristine, cisplatin, and oxaliplatin in cultured human glioblastoma (8MGBA and U251MG) and non-tumor (Lep-3) cells.
