# Supplementary material for: {ZnII2} and {ZnIIAuI} Metal Complexes with Schiff Base Ligands as Potential Antitumor Agents Against Human Glioblastoma Multiforme Cells
Source: Molecules. 2026 Jan 1;31(1):173. doi: 10.3390/molecules31010173 (PMC12787429; doi:10.3390/molecules31010173)
Supplement: Supplementary file 1 [file molecules-31-00173-s001.zip › molecules-4043229-supplementary.pdf]

Electronic Supplementary Information

{Zn^{II}_2} and {$Zn^{II}Au^{I}$} Metal Complexes with Schiff Base Ligands as Potential Antitumor Agents Against Human Glioblastoma Multiforme Cells

Lora Dyakova [1,†], Tanya Zhivkova [2], Abedulkadir Abudalleh [2], Daniela C. Culita [3,†], Teodora Mocanu [3], Augustin M. Madalan [4], Anamaria Hanganu [4,5], Gabriela Marinescu [3,*], Emanuil Naydenov [6] and Radostina Alexandrova [2,*]

[1] Institute of Neurobiology, Bulgarian Academy of Sciences, Acad. Georgi Bonchev Str., Bl. 23, Sofia 1113, Bulgaria; sigma13@abv.bg (L.D.)
[2] Institute of Experimental Morphology, Pathology and Anthropology with Museum, Bulgarian Academy of Sciences, Acad. Georgi Bonchev Str., Bl. 25, Sofia 1113, Bulgaria; tani413@abv.bg (T.Z.); alkader78mah@yahoo.com (A.A.); rialexandrova@hotmail.com (R.A.)
[3] Ilie Murgulescu Institute of Physical Chemistry, Romanian Academy, 202 Splaiul Independentei, 060021 Bucharest, Romania; dculita@icf.ro (D.C.C.); tmocanu@icf.ro (T.M.); gmarinescu@icf.ro (G.M.)
[4] Department of Inorganic Chemistry, Organic Chemistry, Biochemistry and Catalysis, Faculty of Chemistry, University of Bucharest, 90–92 Panduri St., 050663 Bucharest, Romania; augustin.madalan@chimie.unibuc.ro (A.M.M); anamaria_hanganu@yahoo.com (A.H.)
[5] "C.D. Nenitzescu" Institute of Organic and Supramolecular Chemistry of the Romanian Academy, 202B Splaiul Independentei, 060023 Bucharest, Romania; anamaria_hanganu@yahoo.com (A.H.)
[6] Department of Neurosurgery, University Hospital St. Ivan Rilski, Blvd. "Acad. Ivan Geshov" 15, Sofia, Bulgaria; emanuilis@abv.bg (E.N.)

*Correspondence: gmarinescu@icf.ro (G.M.); rialexandrova@hotmail.com (R.A.)
† These authors contributed equally to this work.

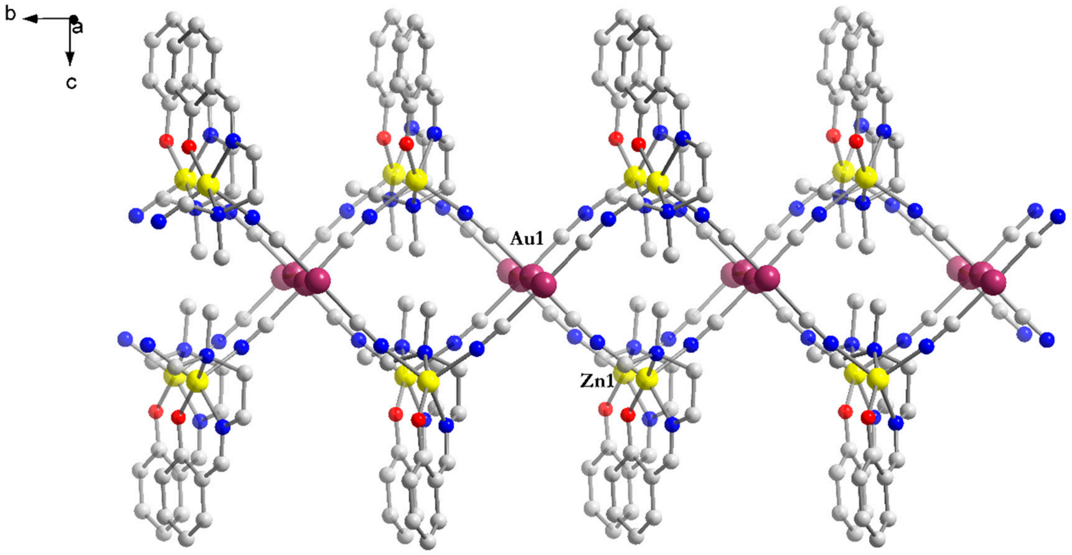

Figure S1. View of packing diagram of **ZnSaldmenAu** along the crystallographic *a* axis.

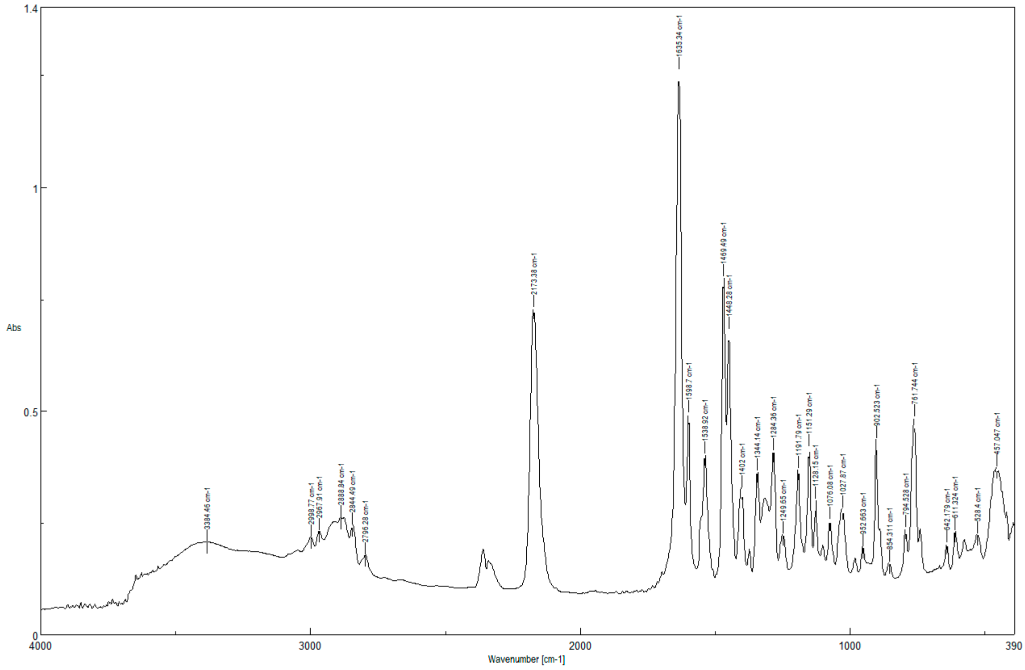

Figure S2. The FT/IR spectrum of **ZnSaldmenAu**.

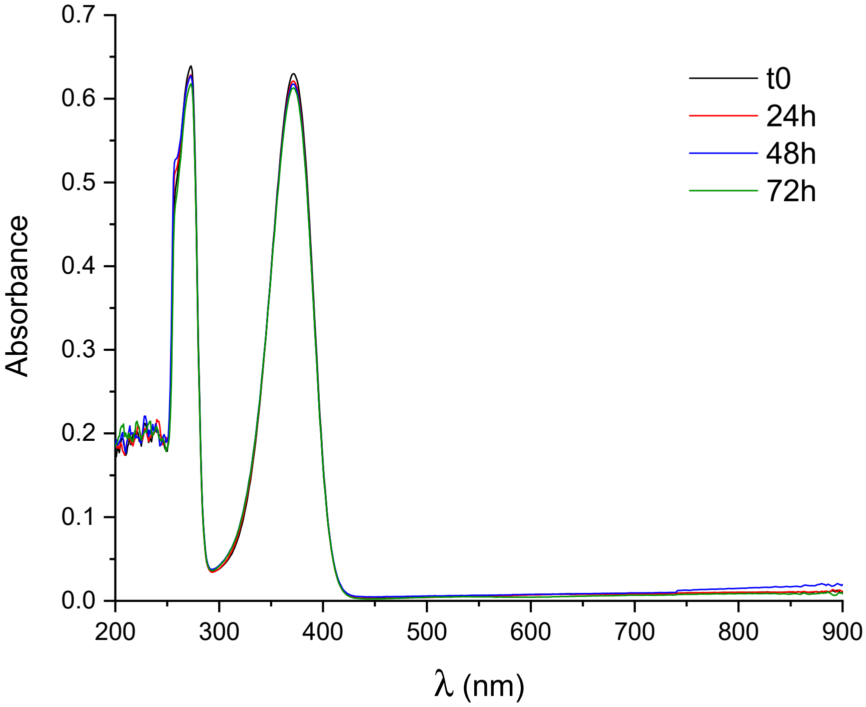

Figure S3. UV-Vis spectra of **ZnSaldmenAu** in DMSO solution at t_0 and after 24, 48, and 72 h.

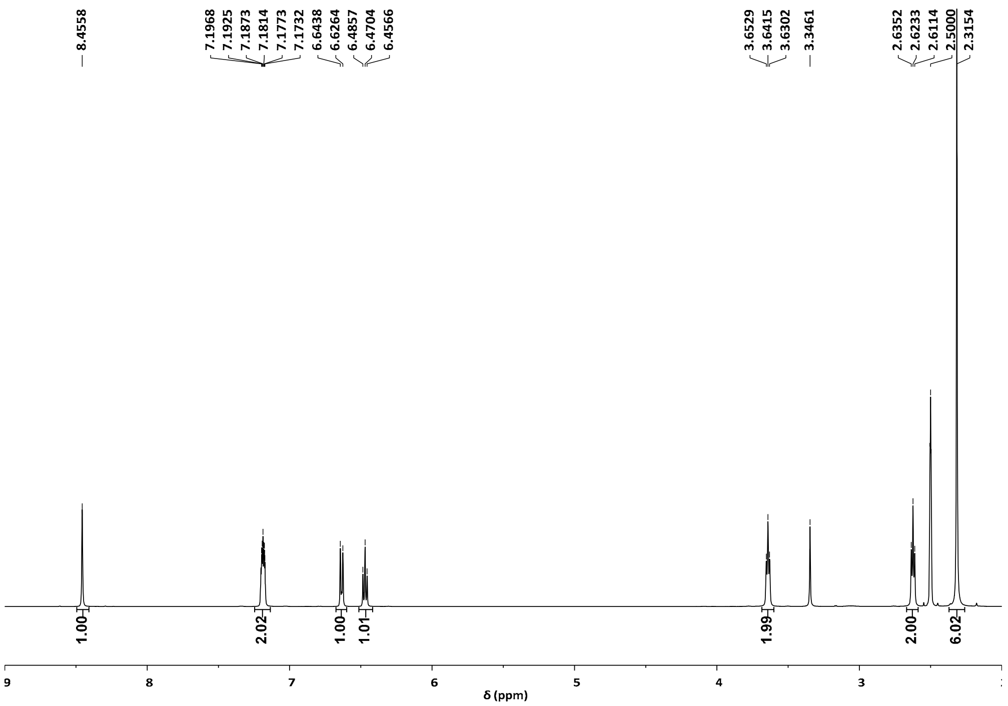

(a)

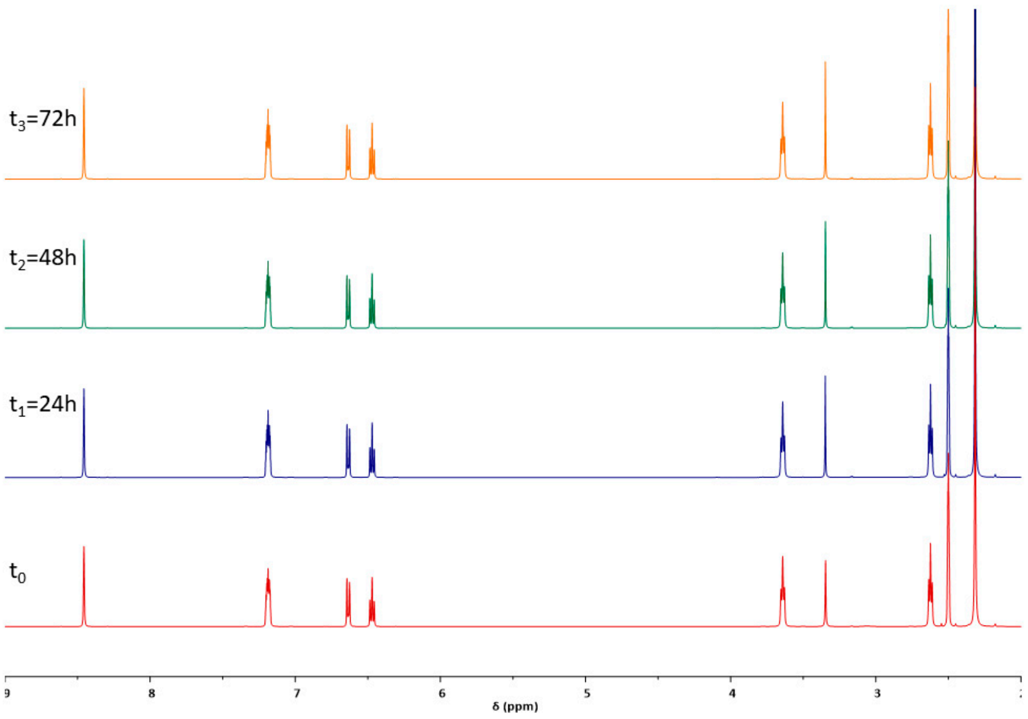

(b)

Figure S4. ^{1}H NMR (DMSO-d6) spectra of **ZnSaldmenAu**: (a) initial spectrum and (b) recorded at t_0 and after 24, 48, and 72 h.

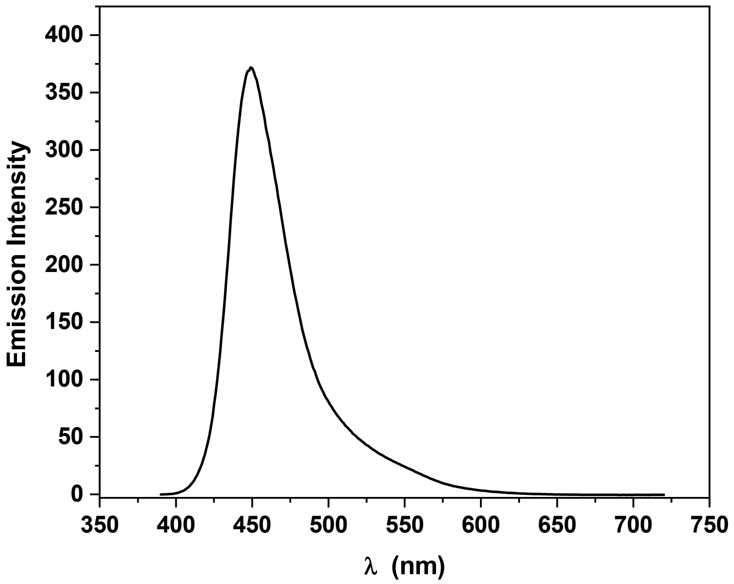

Figure S5. Solid-state emission spectrum of **ZnSaldmenAu,** at room temperature (λ_{exc} = 370 nm).

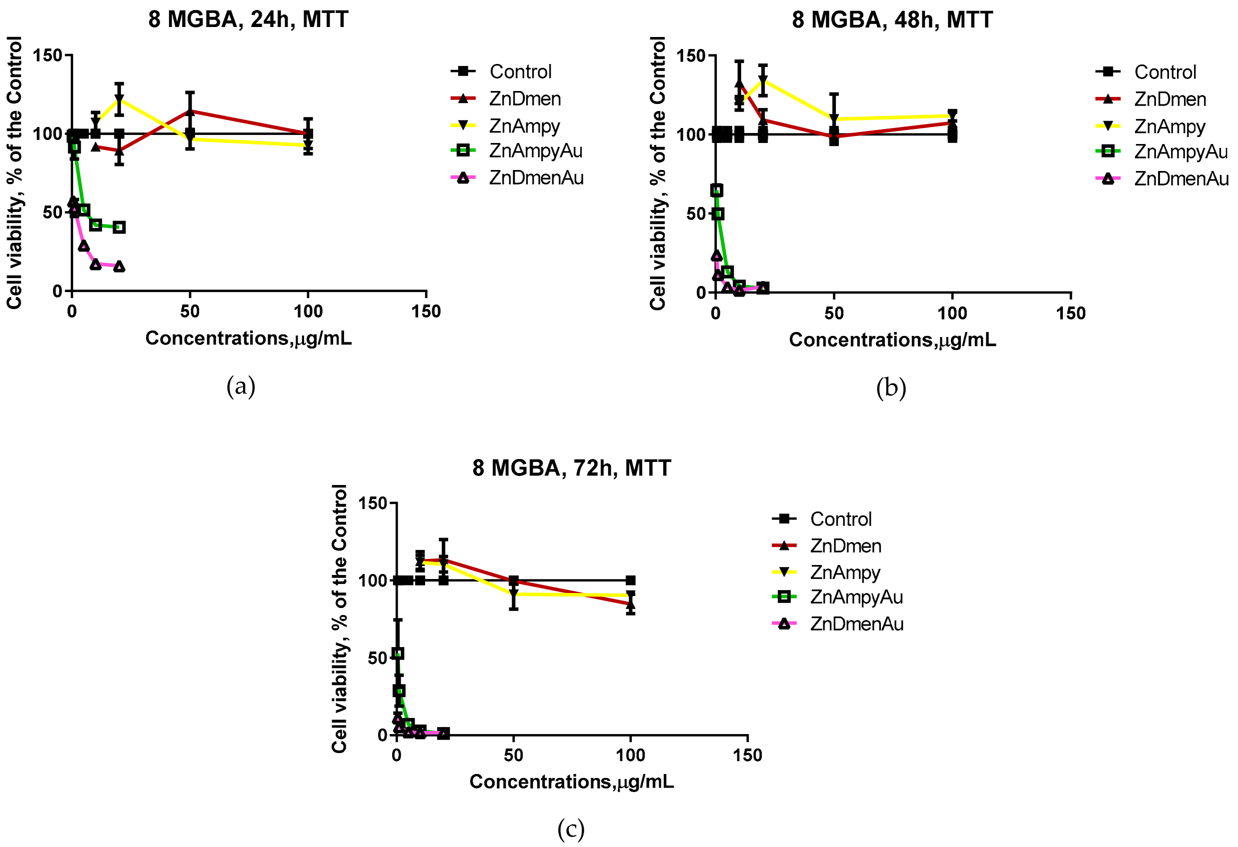

Figure S6. Cytotoxic activity of **ZnAmpy**, **ZnDmen**, **ZnAmpyAu**, and **ZnDmenAu** in human 8MGBA cells. The cell viability and proliferation were determined by MTT test after 24 (a), 48 (b) and 72 h (c) treatment periods.

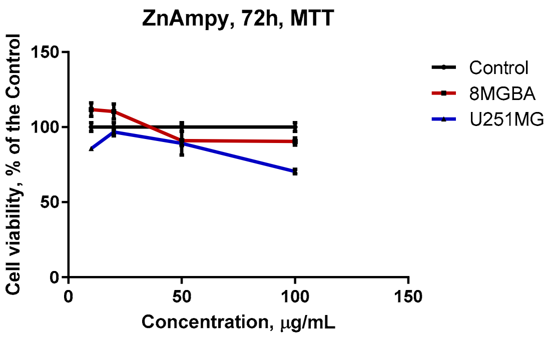

(a)

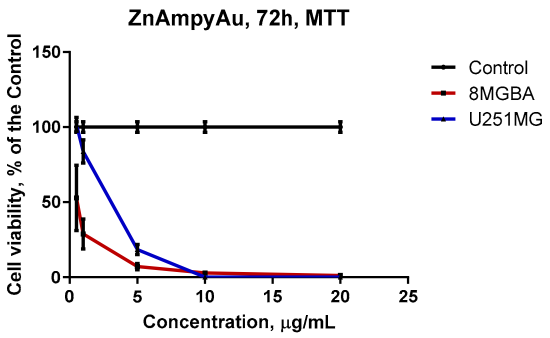

(c)

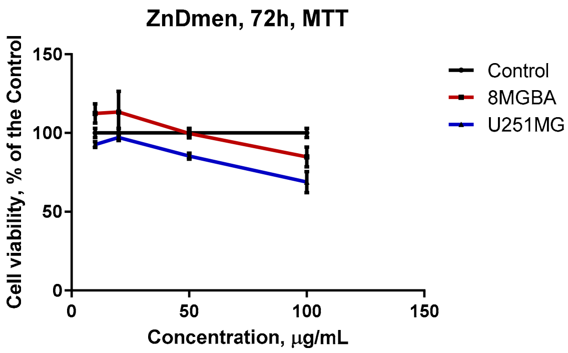

(b)

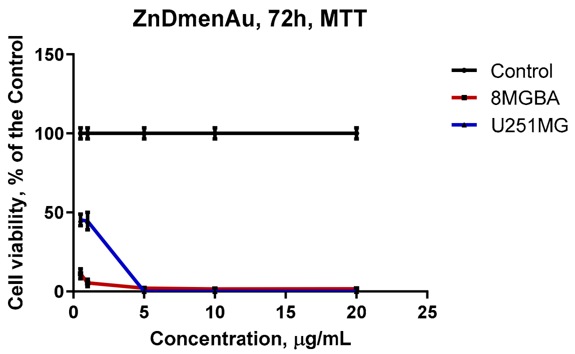

(d)

Figure S7. Cytotoxic activity of **ZnAmpy** (a), **ZnDmen** (b), **ZnAmpyAu** (c), and **ZnDmenAu (**d**)** in human 8MGBA and U251MG cells. Cell viability and proliferation were determined by MTT test after a 72 h treatment period.

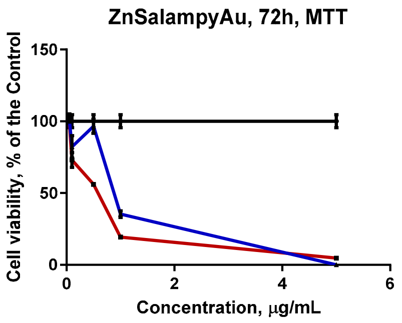

(a)

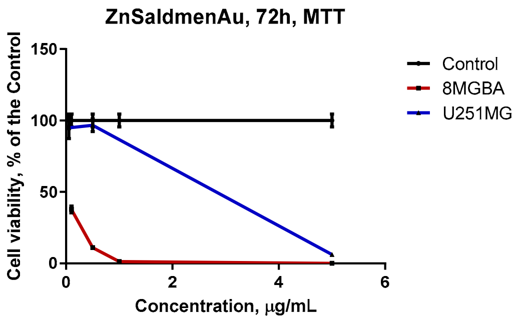

(b)

Figure S8. Cytotoxic activity of **ZnSalampyAu** (a) and **ZnSaldmenAu** (b) in human 8MGBA and U251MG glioblastoma cells. The cell viability and proliferation were determined by MTT test after a 72 h treatment period.

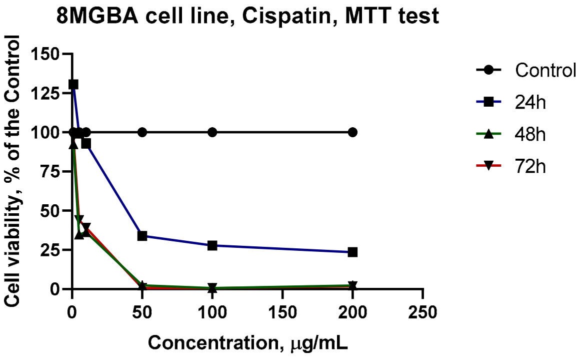

(a)

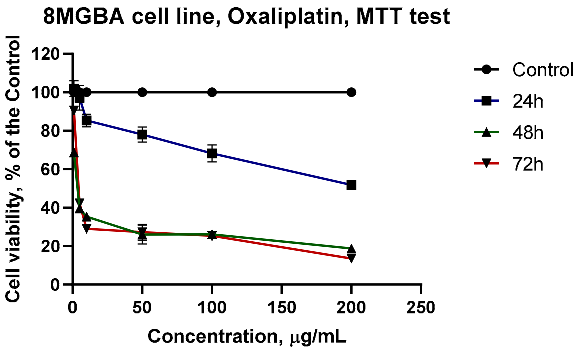

(b)

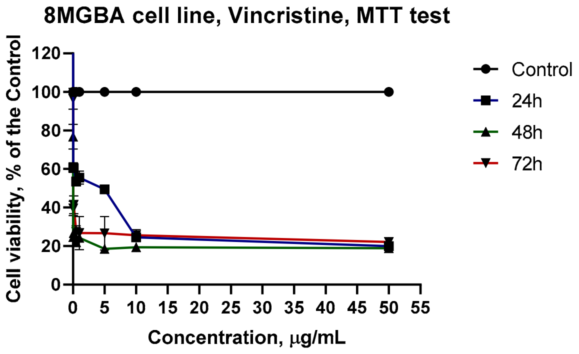

(c)

Figure S9. Glioblastoma multiforme cells (8MGBA permanent cell line) treated with Cisplatin (a), Oxaliplatin (b), and Vincristine (c) for 24, 48 and 72 h. Cell viability was evaluated by MTT test.

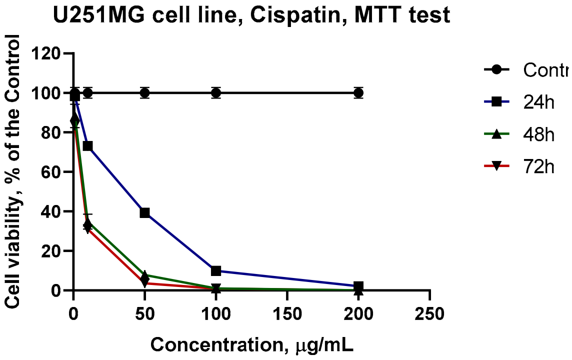

(a)

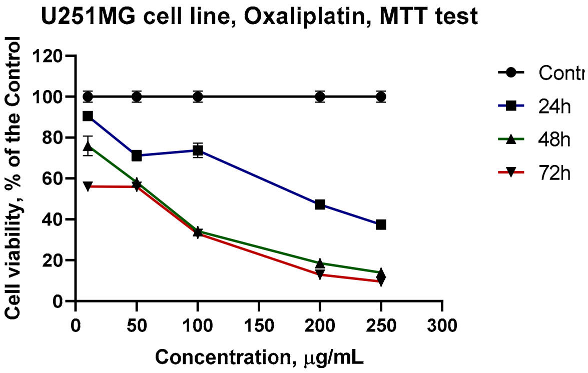

(b)

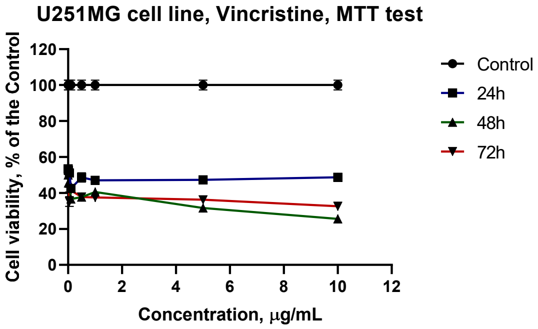

(c)

Figure S10. Glioblastoma multiforme cells (U251MG permanent cell line) treated with Cisplatin (a), Oxaliplatin (b), and Vincristine (c) for 24, 48, and 72 h. Cell viability was evaluated by MTT test.

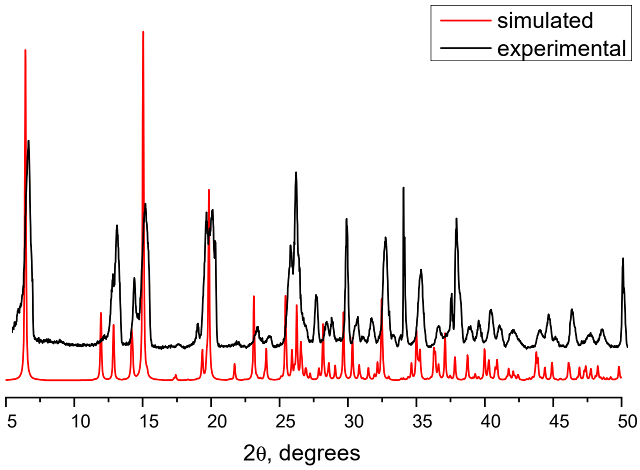

Figure S11. X-ray powder diffractograms of **ZnSaldmenAu** simulated from the SC-XRD data (red line) and experimental PXRD pattern (black line).

Table S1. Crystallographic details of data collection and structure refinement parameters for compound **ZnSaldmenAu**.

Compounds	**ZnSaldmenAu**
Chemical formula	$C_{13}H_{15}ZnN_4OAu$
FW (g mol^{-1})	505.63
Temperature, (K)	293(2)
Crystal system	Orthorhombic
Space group	Pbca
a (Å)	6.9991(3)
b (Å)	15.3714(7)
c (Å)	27.4926(12)
α(°)	90
β(°)	90
χ(°)	90
V (Å^3)	2957.8(2)
Z	8
D_c (g cm^{-3})	2.271
μ (mm^{-1})	11.530
F(000)	1904.0
Goodness-of-fit on F^2	1.077
Final R_1, wR_2 [$I > 2\sigma(I)$]	0.0502, 0.1234
R_1, wR_2(all data)	0.0623, 0.1309
Largest diff. peak and hole (eÅ^{-3})	2.77, -1.30

Table S2. Selected geometric parameters: bonds (Å) and angles (°) in compound **ZnSaldmenAu**.

**ZnSaldmenAu**	
Bond (Å)	Angles (°)
Zn1-N1 = 2.039(7)	N2-Zn1-N1 = 77.7(3)
Zn1-N2 = 2.221(7)	N3-Zn1-N1 = 145.9(3)
Zn1-N3 = 2.093(7)	N1-Zn1-N4[a] = 108.6(3)
Zn1-O1 = 1.954(6)	N2-Zn1-N3 = 87.9(3)
Zn1-N4[a] = 2.072(7)	N2-Zn1-N4[a] = 93.0(3)
Au1-C13 = 1.993(8)	N3-Zn1-N4[a] = 102.8(3)
Au1-C12 = 1.988(9)	N1-Zn1-O1 = 90.5(3)
	N3-Zn1-O1 = 92.7(3)
	N2-Zn1-O1 = 159.2(3)
	O1-Zn1-N4[a] = 107.2(3)
	C12-Au1-C13 = 178.3(3)
a = 1-x, -0.5+y, 0.5-z	

Table S3. Cytotoxic activity CC_{50}* and CC_{90}** (in parentheses) (µM) of conventional antitumor agents Vincristine, Cisplatin, and Oxaliplatin in cultured human glioblastoma (8MGBA and U251MG) and non-tumor (Lep-3) cells.

**8MGBA**			
	**24 h**	**48h**	**72h**
**CisPlatin**	11.86*± 2.4 (> 60.22 ± 4.1)**	1.18 ± 1.9 (12.39 ± 3.2)	1.36 ± 1.4 (12.09± 3.6)
**Oxaliplatin**	≥ 79.46 ± 3.8	1.41 ± 1.6 (> 79.46 ± 3.9)	1.73 ± 1.2 (> 79.46 ± 4.3)
**Vincristine**	3.81 ± 1.9 (> 41.25 ± 3.8)	0.02 ± 1.1 (> 41.25 ± 3.2)	0.03± 0.9 (> 41.25± 3.6)
**U251MG**			
	**24 h**	**48h**	**72h**
**CisPlatin**	11.18 ± 1.2 (30.08 ± 3.5)	2.28 ± 1.2 (14.17 ± 2.2)	2.07 ± 1.8 (12.26 ± 2.0)
**Oxaliplatin**	75.35 ± 2.4 (> 99.32 ± 2.6)	26.60 ± 2.9(> 99.32 ± 3.4)	25.08 ± 2.1 (96.88± 4.1)
**Vincristine**	0.05 ± 2.0 (> 8.25 ± 2.0)	0.004 ± 1.3 (> 8.25 ± 2.5)	0.007 ± 0.9 (> 8.25 ± 2.5)
**Lep-3**			
	**24h**	**48h**	**72h**
**CisPlatin**	-	27.60 ± 2.4 (93.56 ± 3.1)	1.63 ± 2.0 (29.66 ± 3.0)
**Oxaliplatin**	174.46 ± 2.7(-)	76.82 ± 3.2 (251.71 ± 4.7)	2.44 ± 1.6 (125.85 ± 4.1)
**Vincristine**	n.da.	n.da.	n.da.

"-"- the concentration cannot be calculated, because cell viability is > 50%; "n.da."- no data.
